# Structural equation modeling of tourist loyalty in Tianshui, China: Perceived service quality, perceived social value, and crowding effects

**DOI:** 10.1371/journal.pone.0357597

**Published:** 2026-10-07

**Authors:** Shuang An, Dan-ni Gu, He-jun Huang, Jun-qiang Luo, Rong-rong Liu

**Affiliations:** 1 College of Earth and Environmental Sciences, Lanzhou University, Lanzhou, Gansu, China; 2 College of Agriculture and Forestry Economics Management, Lanzhou University of Finance and Economics, Lanzhou, Gansu, China; 3 School of Economics, Lanzhou University of Finance and Economics, Lanzhou, Gansu, China; Sichuan Agricultural University, CHINA

## Abstract

This study explores how the unique perceptions of Internet-Famous destinations affect tourist loyalty and its underlying mechanisms. Based on 375 valid questionnaires collected in Tianshui, an “Internet-Famous” city, this study constructs a structural equation model to test the main effects, mediating effects, and moderating effects, thereby verifying the hypothesized relationships. The results show that perceived service quality is a direct predictor of tourist loyalty and plays a full mediating role between perceived social value and loyalty. Perceived crowding is negatively correlated with perceived service quality, positively correlated with perceived social value, and exhibits a U-shaped non-linear relationship with loyalty. The perceived playfulness of online information negatively moderates the relationship between perceived service quality and loyalty. These findings provide managerial implications for the sustainable development strategies of Internet-Famous destinations.

## Introduction

In the post-pandemic era, a new trend has emerged in Chinese tourism: once-obscure locations are achieving overnight fame through viral short videos. These Internet-Famous destinations capture widespread public attention and draw massive influxes of visitors [1]. An Internet-Famous destination refers to a city that gains immense popularity and becomes a viral sensation through social media platforms like Douyin and Xiaohongshu [2]. Notable examples include the Zibo BBQ craze, Harbin’s “Ice and Snow” season, and the Tianshui “Malatang” trend. While these viral spikes trigger a short-term surge in tourism revenue, they also present a new challenge: **how to transform transient popularity into sustained tourist interest** [3].

Enhancing Loyalty is a key mechanism for converting fleeting attention into lasting interest. Visitor loyalty is defined as the tendency of visitors to revisit a destination or recommend it to others [4]. Research indicates that increasing loyalty not only stabilizes tourist flow and promotes a positive destination image but is also vital for the long-term viability of Internet-Famous destinations [5,6]. Among the antecedents of loyalty, visitor perception has been identified as a primary predictor [7–10]. However, in social media-driven tourism, the viral nature of short videos allows destinations to gain immense reputation and novelty overnight; it also means visitors form an image of the destination before they even arrive. This shift alters the dimensions of visitor perception. It is no longer determined solely by the destination’s service quality, but is also influenced by the social prestige gained from “checking in,” the authenticity of the “viral” atmosphere, the Perceived Crowding caused by mass influxes, active sharing on social platforms, and the psychological benefits of digital experiences [11–15]. Whether and how these new, digitally-mediated factors ultimately influence and translate into visitor loyalty remains under-explored in empirical research. Therefore, taking Tianshui—a prominent Internet-Famous destination in China—as a case study, this paper explores how the unique perceptions of such destinations impact loyalty and the underlying mechanisms involved. Our goal is to provide foundational evidence for destination marketing strategies driven by social media.

### Research framework

#### Loyalty.

The construct of Loyalty originates from consumer behavior research and refers to a consumer’s psychological commitment to continue consuming a specific product or service [4]. Empirical studies show that consumer loyalty is a vital driver of corporate performance growth [16]. Compared to general consumer loyalty, the connotation of tourist loyalty is more diverse and is typically categorized into attitudinal loyalty, behavioral loyalty, and composite loyalty. Attitudinal loyalty reflects a tourist’s willingness to positively recommend a destination to others; behavioral loyalty is manifested as the intention to revisit or repurchase tourism services; and composite loyalty is the organic integration of both [17]. Currently, composite loyalty has become the mainstream operational definition for measuring tourist loyalty in tourism research [18,19]. In a social media-driven tourism environment, loyal tourists do more than contribute economic value through repeat visits; their spontaneous positive word-of-mouth effectively reduces the perceived risk for potential visitors and dispels doubts regarding “marketing filters” [20]. This trust-based loyal relationship is crucial for destination image restoration and long-term development. Accordingly, this study measures the Loyalty of tourists at Internet-Famous destinations through the dual dimensions of behavior and attitude.

### Perceived service quality

Perceived service quality is a subjective evaluation of service performance, reflecting a consumer’s long-term and holistic perception of services as well as their overall attitude toward the service provider [21,22]. As a vital antecedent of Loyalty, perceived service quality effectively explains tourist emotions and predicts behavioral intentions such as revisiting and recommending [23]. Regarding measurement, early studies frequently employed Gap Theory, defining perceived service quality as a comparison between consumer service expectations (Expectations) and actual perceptions (Perceptions), measured via the SERVQUAL five-dimensional framework [24]. Subsequent research has gradually confirmed that performance-based measurement methods offer superior validity and have since become the mainstream approach [21]. With the evolution of tourism research, scholars have begun to flexibly reconstruct measurement dimensions based on specific contexts, integrating factors such as food quality, service-scape, aesthetic atmosphere, convenience, and safety into assessment systems [25]. For Internet-Famous destinations, the service experience is often composed of both online digital landscapes and offline physical services.For Internet-Famous destinations, tourists have already learned information about Internet-Famous communication subjects (such as Zibo barbecue, Tianshui Malatang, etc.) in advance through social media, forming expectations of the tourism experience. According to Expectation-Confirmation Theory, when tourists’ actual experiences at the destination match or even exceed online information, their satisfaction increases significantly [26,27]. Therefore, the perceived service quality of Internet-Famous destinations includes not only perceptions of attraction playfulness and basic tourism services such as accommodation and transportation, but also perceptions of the quality of Internet-Famous communication subjects, which is a key feature distinguishing the perceived service quality of Internet-Famous destinations from that of traditional destinations.

### Perceived social value

Extensive research has confirmed that perceived value is a key driver of Loyalty [28–30]. As a core dimension of perceived value, Perceived Social Value is described in the classic PERVAL scale through sub-dimensions such as the utility derived from enhancing image, identity, and social status, as well as gaining social recognition [31]. In the context of Internet-Famous destinations, the measurement of Perceived Social Value is highly correlated with the destination’s online reputation and tourists’ “checking-in” behaviors [32]. Studies indicate that individuals tend to shape an ideal image, earn respect, and accumulate social influence by sharing and showcasing their unique lifestyles [33,34], thereby gaining social capital and further expanding their social circles [35]. With their high exposure and novelty on social media, Internet-Famous destinations provide tourists with an ideal setting to attract social attention [36,37]. Through sharing their check-in activities, tourists gain more social interaction and emotional satisfaction [16,38,39], leading to a value perception of enhanced social prestige and expanded social reach [40], while participating in value co-creation during this process [41]. Unlike traditional destinations, the high Perceived Social Value generated by the short-term online reputation of Internet-Famous destinations is a core characteristic of their perceived value system.

### Perceived crowding

Perceived Crowding is a psychological state resulting from an individual receiving excessive social stimulation (“being crowded” is “receiving excessive stimulation from social sources”) [42]. Stimulus-Overload Theory (SOT) posits that when environmental information and stimuli exceed an individual’s cognitive processing capacity, it triggers cognitive overload and psychological stress [43]. In tourism settings, spatial crowding, traffic congestion, noise interference, and the compression of personal space caused by the concentration of crowds often lead to perceived overload, which in turn induces cognitive fatigue and emotional stress [44–46]. However, while traditional views suggest that the stress generated by crowding inhibits Loyalty [47], the relationship between crowding and Loyalty becomes more complex within the specific context of Internet-Famous destinations. Driven by online reputation, a massive instantaneous influx of tourists makes crowding an objective reality for these destinations. Since tourists often learn about this situation in advance through social media and view it as a symbol of “popularity” and a “viral atmosphere” [48], this prior psychological expectation may offset the negative effects of crowding on tourist perception [49]. Consequently, the relationship between Perceived Crowding and tourist emotions and behaviors becomes increasingly intricate.

### Perceived playfulness

Perceived Playfulness reflects the concentration, immersion, curiosity, and enjoyment experienced by individuals during an interactive process [50]. Empirical studies show that the playfulness of online information is a core intrinsic motivation driving user behavior [51]. In social media-driven tourism, the playfulness of destination-related information is not only a primary reason for achieving “viral spread” [48] but also a key factor influencing tourists’ travel decisions [11]. However, whether the high-frequency exposure accompanying viral transmission affects long-term Loyalty remains to be explored. Hedonic Decline theory suggests that repeated sensory stimulation leads to a decline in an individual’s sense of pleasure [52]. This occurs because consumers tend to internalize past playful experiences as a “reference point” for evaluating future ones, requiring higher-intensity stimuli to maintain the original level of satisfaction [53]. Consequently, the Hedonic Decline caused by repeated stimulation accumulates negative emotions [52], meaning that even currently satisfied customers may hold a negative attitude toward future consumption due to the anticipation of diminishing returns [54]. Therefore, while viral transmission can attract a large number of tourists in the short term, over-saturated information may accelerate consumer Hedonic Decline and could, in the long run, conversely inhibit tourist Loyalty.

### Research hypotheses

Numerous studies have confirmed the positive driving effect of perceived service quality and perceived social value on loyalty [55–60]. This study argues that this relationship also applies to Internet-Famous destinations. Research on tourists’ social media use shows that tourists’ online sharing behavior and the positive feedback received from online audiences can enhance their evaluation of the travel process [61,62], indicating that perceived social value can enhance tourists’ perceived service quality. Accordingly, this study proposes the following hypotheses:

**H1: There is a positive correlation between tourists’ perceived service quality and loyalty at Internet-Famous destinations.**
**H2: There is a positive correlation between tourists’ perceived social value and loyalty at Internet-Famous destinations.**
**H3: There is a positive correlation between tourists’ perceived social value and perceived service quality at Internet-Famous destinations.**

Existing studies have shown that the stress response caused by crowding adversely affects the tourism experience [47], leading tourists to give lower evaluations of perceived service value. However, the bustling atmosphere created by crowding at Internet-Famous destinations satisfies tourists’ conformity tendencies [63], stimulates positive emotions [64,65], and enhances tourists’ perception of the destination’s social influence, which is conducive to the increase of perceived social value. There is currently no consensus on the relationship between crowding and loyalty. Some studies have found that avoidance responses generated by crowding reduce tourists’ revisit intention [66,67], while other studies show that when crowding serves as an attractive social atmosphere, it does not significantly affect tourist behavior [68]. Based on stress overload theory, this study assumes that perceived crowding is negatively correlated with tourist loyalty, and will conduct further analysis based on the test results. In summary, the following hypotheses are proposed:

**H4: There is a negative correlation between tourists’ perceived crowding and perceived service quality at Internet-Famous destinations.**
**H5: There is a positive correlation between tourists’ perceived crowding and perceived social value at Internet-Famous destinations.**
**H6: There is a negative correlation between tourists’ perceived crowding and loyalty at Internet-Famous destinations.**

This study posits that the perceived playfulness of online content plays a negative moderating role in the path between perceived service quality and loyalty. For Internet-Famous destinations, the playfulness of online information serves as the core driving force for its viral dissemination [69]. For users with high perceived playfulness, the interest-driven recommendation mechanisms of video platforms repeatedly push destination information [70]. Influenced by hedonic adaptation, the incremental hedonic benefits that should have been generated by offline services are consumed in advance by online stimulation. Consequently, tourists enter a state of perceptual saturation [71], which reduces the utility of perceived service quality in driving loyalty. Conversely, for tourists with low perceived playfulness, because they are not frequently exposed to playful destination information, the driving effect of perceived service quality on loyalty is not significantly affected. Based on this, this study proposes the following hypothesis:

**H7: At Internet-famous destinations, tourists’ perceived playfulness of online content negatively moderates the relationship between perceived service quality and loyalty.**

It should be noted that all the above hypotheses are alternative hypotheses.

Based on the hypotheses proposed in this study, we have constructed a theoretical model linking the perception of value at Internet-famous destinations with tourist loyalty, as illustrated in Fig 1.

**Fig 1:**
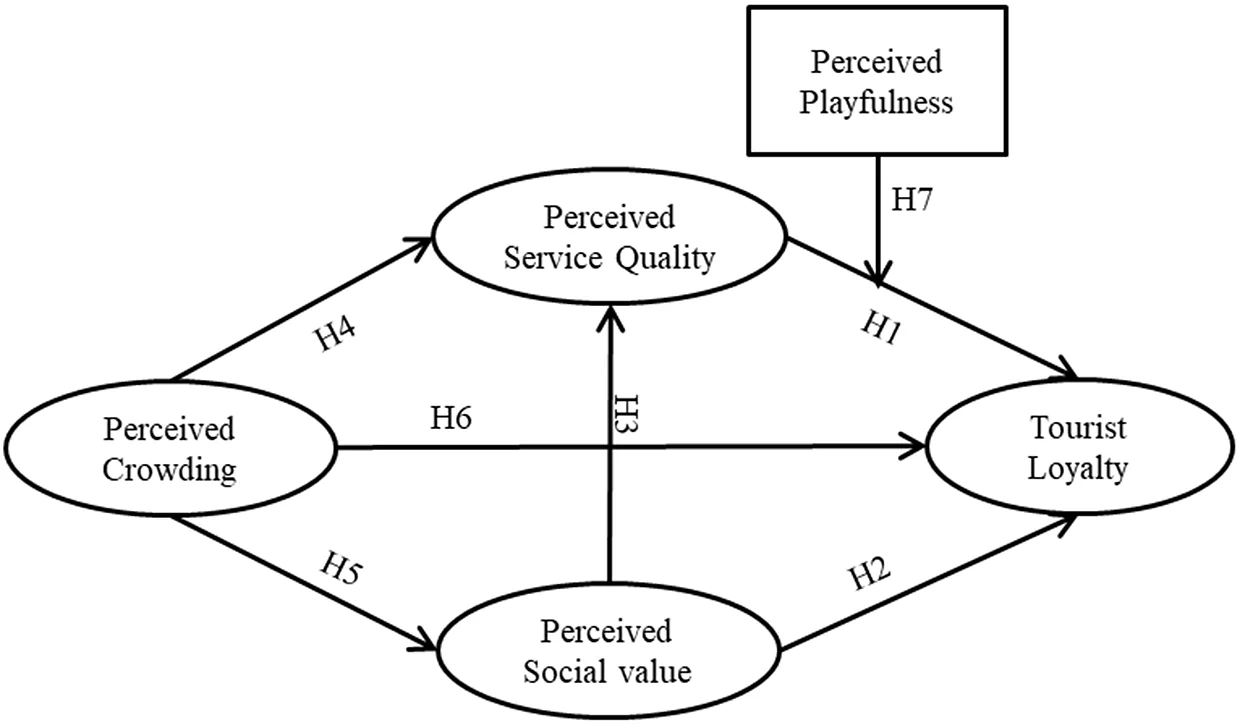
Theoretical model of the research hypotheses.

## Materials and methods

### Study area

Tianshui is located in the southeastern part of Gansu Province, China. It is a historical and cultural city with over 4,000 years of recorded civilization and abundant tourism resources. In early March 2024, a short video showcasing Tianshui “Malatang” (a local street snack) went viral on Chinese social media platforms, transforming the city into an Internet-famous destination. This led to a significant influx of food bloggers and tourists seeking to experience the culinary offerings depicted in the video.

The popularity trajectory of Tianshui mirrors that of other Chinese viral destinations, such as Zibo, Harbin, and Huaihua, following a cycle of “online viral transmission, offline tourist surge, online heat decay, and return to market normalcy.” Baidu Index monitoring indicated that online searches for “Tianshui Malatang” began to increase on March 14, 2024, and reached a peak of 14,554 on March 18. This was followed by a fluctuating decline in a long-tail pattern, returning to 1,233 by May 5, with the entire viral cycle lasting approximately two months. Official statistics showed that during this period, the cumulative number of tourists in Tianshui increased by 35.69% year-on-year [72]. This phenomenon provides a suitable empirical context for studying tourist loyalty driven by social media.

### Data collection

The research data for this study were collected through a cross-sectional survey of tourists visiting Tianshui during a period of viral popularity. The survey adopted a convenience sampling method. During the Qingming Festival holiday (April 4–6, 2024), tourists were intercepted at major scenic spots and popular “check-in” locations in Tianshui City. Trained interviewers read the questionnaire items to respondents and recorded their answers via the “Sojump” WeChat mini-program. A total of 400 questionnaires were completed. After excluding questionnaires from local residents and those with careless responses (e.g., selecting the same option for multiple consecutive questions, excessively short completion times), a total of 375 eligible questionnaires were obtained, accounting for 93.75% of the initial sample. Prior to the formal survey, a pre-survey was conducted to test the clarity of the questionnaire items.

This study strictly follows scientific research ethics codes. Training was conducted for investigators in advance, the content of which included unifying investigation standards; standardizing interception timing, interception targets, and standing positions; mastering communication skills and contact etiquette; and strictly enforcing investigation discipline and ethics training, etc. The investigation adopts the principle of voluntary participation. Before the investigation begins, investigators will explain the purpose, content, and data protection measures of this survey to the respondents, and it can only begin after obtaining consent; respondents can terminate participation at any time, and questionnaires terminated midway shall be deemed invalid. All investigations are conducted anonymously, without collecting any identifiable personal identity information of the respondents, therefore all informed consent is obtained in the form of oral consent. Minors accept interviews only when accompanied by parents or guardians, and must obtain the oral consent of the parents. The questionnaire content centers around tourists’ tourism experience, without any questions involving privacy, sensitivity, or offensiveness, and does not conduct psychological guidance. After the investigation is completed, each respondent is presented with a postcard as a thank you. This study obtained an ethics review waiver from the Science and Technology Department of Lanzhou University of Finance and Economics.

### Variable measurement

The measurement scales for this study were adapted from established research and refined through localized and contextual adjustments based on pre-survey feedback. Five formal research constructs were developed to examine the factors influencing loyalty toward Internet-famous destinations. Based on the results of exploratory and confirmatory factor analyses, items with factor loadings below 0.5 were removed, resulting in a final measurement system comprising 13 items. All constructs were measured using a five-point Likert scale, ranging from 1 (strongly disagree) to 5 (strongly agree). The Cronbach’s alpha coefficients for the four primary dimensions (excluding the moderating variable) ranged from 0.699 to 0.855, meeting or approaching the threshold of 0.70. The overall scale demonstrated good internal consistency with a Cronbach’s alpha coefficient of 0.860 [73].

Tourist loyalty was measured using three indicators: “recommendation intention” for attitudinal loyalty and “revisit intention” for behavioral loyalty [10]. Additionally, a composite loyalty indicator, “revisiting with family and friends,” was developed. This indicator reflects not only the individual’s intention to return but also their social motivation to recommend the destination to others, representing a higher level of destination attachment and behavioral tendency. It should be noted that as the data were collected from on-site tourists, the loyalty measured in this study refers to “loyalty intentions” rather than actual loyalty behavior.

To measure perceived service quality, this study followed the construction method of the HOLSAT scale [74]. Four indicators were selected to measure tourists’ objective perception of service quality: “food variety,” “cultural heritage of attractions,” “urban environment,” and “supporting services.”

For perceived social value, the study referred to the PERVAL scale [31]. Three indicators were employed: “becoming more popular among friends,” “increasing attention on social media (e.g., Moments),” and “making more friends.”

The measurement of perceived crowding was adjusted based on pre-survey feedback. Since crowding at viral destinations is an objective reality, tourists’ basic perceptions (e.g., “I feel there are too many people”) showed high convergence, leading to a lack of statistical variance and difficulty in predicting the impact of emotional stress on subsequent constructs. Consequently, this study utilized tourists’ perception of crowding stress as a proxy indicator to measure perceived overload and emotional response. According to Cognitive Appraisal Theory (CAT) [75], an individual’s response to a stressful event comprises two dimensions: internal emotional experience and external coping tendencies. These two elements, as products of the same appraisal process, exhibit high psychological coherence [76]. Therefore, two items were selected for measurement: “Crowding affected my travel experience,” which focuses on the interference of stress with individual goals (reflecting internal loss-benefit appraisal), and “One should avoid hotspot areas and return later,” which emphasizes stress-driven avoidance intentions (reflecting external coping tendencies). Together, these items represent the comprehensive stress response of tourists in crowded environments from both cognitive and behavioral perspectives [77], thereby reflecting deeper perceptions of crowding and ensuring statistical differentiation.

The perceived playfulness of online information was measured using the item: “I find the video’s comment section very interesting.” According to Moon & Kim (2001), perceived playfulness is defined as an intrinsic motivation reflecting a “flow experience” during user-system interaction [78], which constitutes an intuitive evaluation. Video comment sections frequently serve as hubs for secondary creation and primary sources of Internet memes; the abundant humor and social resonance found therein can generate significant emotional pleasure and sensory satisfaction [79], aligning with the hedonic nature of this construct. In social media-driven tourism contexts, following viral videos is a fundamental behavior of tourists, providing them with a clear cognitive basis to assess the interest level of comment sections. Following Bergkvist et al. (2007), for well-defined variables with concrete attributes, a single-item measure can effectively capture respondents’ authentic perceptions [80]. Therefore, this study employed a single-item indicator for the moderating variable to meet the statistical requirements for moderation analysis, treating it as a fixed error term to examine its moderating effect.

## Results

Among the respondents, young people aged 18–25 years constituted the vast majority (58.7%), and 72.0% held a bachelor’s degree or junior college diploma. More than 50% of the respondents reported a monthly income below 3,000 RMB. The proportion of female participants was higher than that of males (55.7% vs. 44.3%). Detailed demographic data are presented in Table 1.

**Table 1:** Demographic profile of participants.

Variables	frequency	Percent (%)
**Sex**		
Male	166	44.3
Female	209	55.7
**Age**		
Under 18	22	5.9
18-25 years	220	58.7
26-30 years	73	19.5
31-40 years	41	10.9
41-50 years	13	3.5
51-60 years	5	1.3
61 years and over	1	0.3
**Educational level**		
Junior high school and below	40	10.7
High school or junior college	33	8.8
Bachelor’s degree or college diploma	270	72.0
Master’s degree and above	32	8.5
**Monthly salary**		
Under 3000 yuan	192	51.2
3000-5999 yuan	117	31.2
6000-8999 yuan	46	12.3
9000 yuan and over	20	5.3

Overall, culinary tourists provided favorable evaluations of Tianshui’s products and services. each item score exceeded the scale midpoint of 3, with all items pertaining to “experience quality” and “loyalty” scoring above 4. The highest-scoring items included “The food in Tianshui is very diverse” (M = 4.31, SD = 0.761), “Tianshui’s attractions possess profound cultural heritage” (M = 4.30, SD = 0.786), and “I would recommend visiting Tianshui to my family and friends” (M = 4.19, SD = 0.797). These results indicate a positive overall travel experience and high levels of tourist loyalty. The item with the lowest mean and the greatest perceptual variance was “The degree of crowding in Tianshui affected my travel experience” (M = 3.37, SD = 1.17), suggesting significant divergence in tourists’ evaluations of crowding. Furthermore, 84.8% of respondents identified the enticing *Malatang* (spicy hot pot) featured in short videos as their primary motivation for visiting Tianshui, and 81.4% were aware of the city’s crowded conditions prior to departure. This confirms that viral videos were the principal driver of tourist arrivals and that visitors possessed clear antecedent awareness of the destination’s crowding.

### Measurement model testing

Common method bias (CMB) was first examined. A Harman’s single-factor test was conducted as a preliminary assessment. The unrotated exploratory factor analysis (EFA) of all scale items revealed that the first principal factor explained 41.593% of the variance. As this slightly exceeded the 40% threshold, it suggested the potential presence of common method variance (CMV). Following the recommendations of Tang et al. (2020) [81], the CFA marker variable technique was employed to further verify the severity of this bias. A marker variable (M), unrelated to the research variables, was added to the CFA model to construct four sequential models: the baseline model (assuming no CMV), Model C (assuming CMV with equal influence on all indicators), Model U (assuming CMV with varying influence on all indicators), and Model R (assuming CMV without affecting correlations between variables).

The chi-square differences between these models were estimated and compared. A significant chi-square difference was observed between the baseline model and Model C (Δχ² (Δdf = 1) = 7.762,p = 0.005), indicating the presence of CMV. Furthermore, the difference between Model C and Model U was significant (Δχ²(Δdf = 11) =19.703, p = 0.049), showing that CMV influenced the indicators differently. However, no significant difference was found between Model U and Model R (Δχ² (Δdf = 6)=0.968, p = 0.987). In summary, while slight common method variance exists in the data, it does not constitute a severe common method bias (CMB) and will not substantially interfere with the research results or hypothesis testing. Thus, the data are suitable for subsequent structural model analysis.

Exploratory factor analysis (EFA) and confirmatory factor analysis (CFA) were subsequently performed. To rigorously validate the measurement model (N = 375, missing values < 1%), the total sample was randomly split into two approximately equal halves using SPSS software. The first subsample was used for EFA to identify the factor structure, while the second was used for CFA to confirm the model fit. Finally, a CFA was conducted on the full sample to test the robustness of the results.

EFA was conducted on the first subsample (N = 182) using SPSS 26.0. The KMO value was 0.837, and Bartlett’s test of sphericity yielded χ² = 818.374 (p < 0.001), indicating that the data were suitable for factor analysis. Factors were extracted using the maximum likelihood method. With the eigenvalue-greater-than-1 criterion, the cumulative variance explained was 64.685%. However, the rotated component matrix exhibited cross-loadings and dimension merging, extracting only three factors, which was inconsistent with the theoretical framework of this study. Further assessment of the scree plot revealed that the eigenvalue for a four-dimension structure was 0.970, approaching the critical threshold of 1.0. Considering that a strict reliance on eigenvalues may result in the loss of essential structural information, this study retained the preset four-dimension structure. The construct validity was subsequently subjected to further testing through confirmatory factor analysis (CFA).

CFA was performed on the second subsample (N = 193) using Amos 26.0. The results indicated a good model fit (χ²/df = 1.136, CFI = 0.994, RMSEA = 0.026, SRMR = 0.0348, AIC = 114.550, TLI = 0.992, GFI = 0.957). All standardized factor loadings exceeded 0.60 (p < 0.001), demonstrating that the measurement model was well-specified. Furthermore, all Average Variance Extracted (AVE) values were greater than 0.50, and the square root of the AVE for each factor was higher than its correlation with any other factor. These results confirm that the four-dimension structure possesses robust construct validity, including both convergent and discriminant validity.

A CFA was further conducted using the full sample (N = 375). The results maintained a high level of model fit (χ²/df = 1.444, CFI = 0.988, RMSEA = 0.034, SRMR = 0.0295, AIC = 129.333, TLI = 0.984, GFI = 0.971). The standardized factor loadings for all items ranged from 0.634 to 0.834 (p < 0.001). The Composite Reliability (CR) values ranged from 0.701 to 0.856, and the Average Variance Extracted (AVE) for all constructs exceeded 0.50, providing strong support for convergent validity. Furthermore, the square root of the AVE for each factor was greater than its correlation with any other factor, supporting robust discriminant validity. The factor loadings and convergence results for the full-sample CFA are presented in Table 2, and the discriminant validity results are shown in Table 3.

**Table 2:** Measurement reliability and convergent validity of the scale.

Construct/item	Standardized factor loadings	Cronbach’s α	CR	AVE
**Perceived Service Quality (PSQ)**		0.813	0.813	0.521
X1: Tianshui’s food is very abundant	0.752			
X2: Tianshui’s attractions have a deep cultural heritage	0.725			
X3: Tianshui’s urban environment is very pleasant	0.720			
X4: The quality of tourism services in Tianshui is excellent	0.688			
**Perceived Social Value (PSV)**		0.806	0.828	0.618
X5: This trip can increase my social media attention	0.758			
X6: This trip can make me more popular among my friends	0.883			
X7: This trip can help me make more friends	0.707			
**Perceived Crowding (PC)**		0.699	0.701	0.540
X8: The congestion in Tianshui has impacted my travel experience	0.779			
X9: I should avoid the hotspots and come back to Tianshui after some time	0.688			
**Loyalty**		0.855	0.856	0.666
X10: Given the opportunity, I would visit Tianshui for tourism again next time	0.817			
X11: I would recommend visiting Tianshui to my family and friends	0.834			
X12: I would come to Tianshui for tourism with my family and friends	0.796			

**Table 3:** Discriminant validity.

	AVE	Perceived Service Quality	Perceived Social Value	Perceived Crowding	Loyalty
Perceived Service Quality	0.521	0.722			
Perceived Social Value	0.618	0.699 ***	0.786		
Perceived Crowding	0.540	0.120	0.380***	0.735	
Loyalty	0.666	0.698 ***	0.550***	0.168 *	0.816

significance: *p < .05; **p < .01; ***p < .001

### Hypothesis testing

#### Path analysis.

The structural equation model (SEM) was constructed to test the research hypotheses. The path coefficients and parameters of the main model are detailed in Table 4, and the standardized path coefficients are illustrated in Fig 2. The results show that paths 1, 3, 4, and 5 are significant (reject the null hypothesis), whereas paths 2 and 6 are non-significant (fail to reject the null hypothesis). Therefore, the main model supports hypotheses H1, H3, H4, and H5.

**Table 4:** Results of structural equation model estimation.

Relationship	Unstandardized Coefficients	Standardized Coefficients	C.R.	p
H1 PSQ→Loyalty	0.759	0.610	6.450	<0.001
H2 PSV→Loyalty	0.103	0.103	1.070	0.285
H3 PSV → PSQ	0.617	0.764	9.554	<0.001
H4 PC → PSQ	−0.107	−0.171	−2.504	0.012
H5 PC → PSV	0.296	0.380	4.635	<0.001
H6 PC→Loyalty	0.043	0.056	0.863	0.388

**Fig 2:**
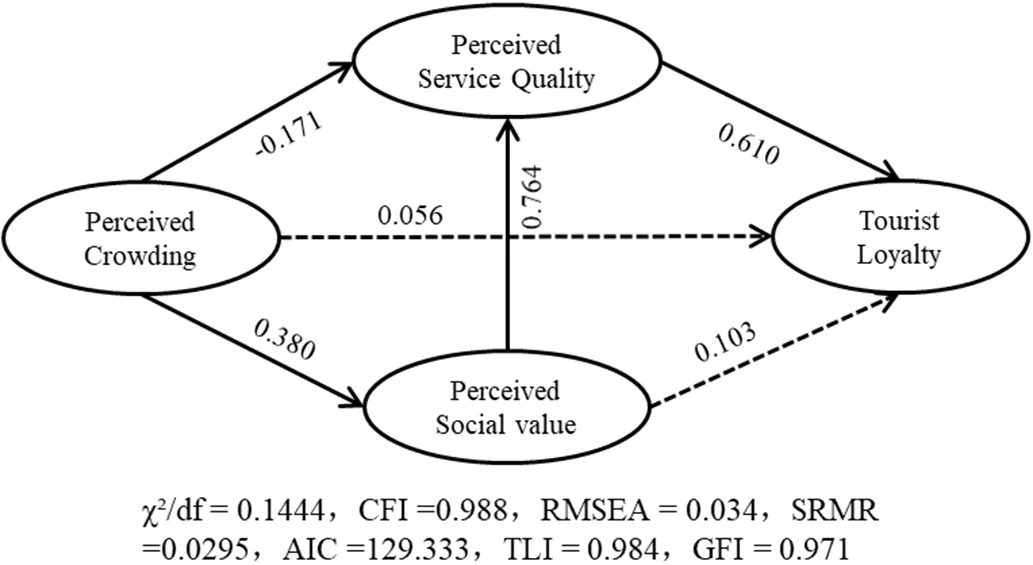
Structural equation model estimation results.

Specifically, for tourists at the destination, perceived service quality has a direct and positive association with loyalty. Although perceived social value is not directly linked to loyalty, it is positively associated with perceived service quality. Furthermore, perceived crowding is negatively associated with perceived service quality but positively associated with perceived social value; it has no significant correlation with loyalty. The results indicate that among the factors examined in this study, perceived service quality is the only direct antecedent of tourist loyalty.

To ensure the statistical robustness of the hypothesized model, a competitive model comparison was conducted. This study primarily tested alternative explanations by reversing the hypothesized path directions. Based on the initial model, the direction of path H3 was reversed to “perceived service quality → perceived social value.” While the global fit indices showed no significant change, the path “ perceived crowding → perceived service quality” lost its significance, indicating a disruption of the model’s internal structural integration and a decline in the identifying power of parameter estimation. Furthermore, reversing the direction of H5 to “perceived social value → perceived crowding” yielded no significant improvement in the overall model fit. When the direction of H6 was reversed to “loyalty → perceived crowding,” the path remained statistically non-significant, suggesting that this causal direction was likewise invalid. In summary, the comparative analysis of competitive models demonstrates that the alternative models offer no advantages in terms of statistical robustness or parameter identification reliability. Given its superior theoretical logic and self-consistency, the initial hypothesized model is preferred over the alternative configurations.

### Mediation effect analysis

Although the direct effect of perceived social value on loyalty (H2) was non-significant, we further explored the possibility that this relationship is fully mediated by perceived service quality. Following the methodology recommended by Wen et al. (2004) [82], we examined the mediating role of perceived service quality between perceived social value and loyalty. The results, as shown in Table 5, indicate that the indirect effect of perceived social value on loyalty through perceived service quality was significant, whereas the direct effect remained non-significant, and the total effect was significant. These findings demonstrate that perceived service quality plays a full mediation role in the relationship between perceived social value and tourist loyalty. In other words, once the mediator (perceived service quality) is introduced, it entirely accounts for the impact of perceived social value on loyalty.

**Table 5:** Results of mediation effect testing.

	Effect	LLCI	ULCI	p	Percent
Indirect effect	0.404	0.253	0.585	0.000	75.65%
Direct effect	0.130	−0.075	0.392	0.211	
Total effect	0.534	0.368	0.724	0.001	

### Moderation effect analysis

The moderating effect of perceived playfulness was subsequently tested. Following the recommendation of Hayduk (1987), the fixed error variance method was employed to construct the moderating variable of perceived playfulness, with its reliability conservatively set at 0.80 [83]. To eliminate multicollinearity and ensure the stability of main effect interpretation, residual centering was applied to the moderating and interaction terms [84]. The processed variables were then integrated into the structural equation model (SEM) for maximum likelihood estimation. The model demonstrated acceptable fit (χ^2^/df = 3.287, CFI = 0.902, RMSEA = 0.078).

Upon adding the interaction term, the coefficient of determination (R²) exhibited a slight decrease (from 0.512 to 0.490, ΔR² = −0.022). Consistent with Kenny (2018), in full-model SEM, R²can be diluted by the accumulation of measurement errors; therefore, we contend that the conclusions regarding the moderating effect are not undermined by this minor fluctuation [85]. Results showed that the path coefficient for the interaction term was significant (β = −0.215, p < 0.001, C.R. = −4.010), confirming the moderating role of perceived playfulness in the relationship between perceived service quality and loyalty, thereby supporting H7. Further validation via simple slope analysis (Fig 3) illustrated that the negative path coefficient of the interaction term (β = −0.215) reflects a “crowding-out” effect: at equivalent levels of perceived service quality, tourists with higher perceived playfulness of online content exhibited lower loyalty compared to others. This suggests that excessive consumption of online playful content may weaken the transformation of perceived service quality into loyalty. The robustness of this finding was further confirmed using hierarchical regression analysis.

**Fig 3:**
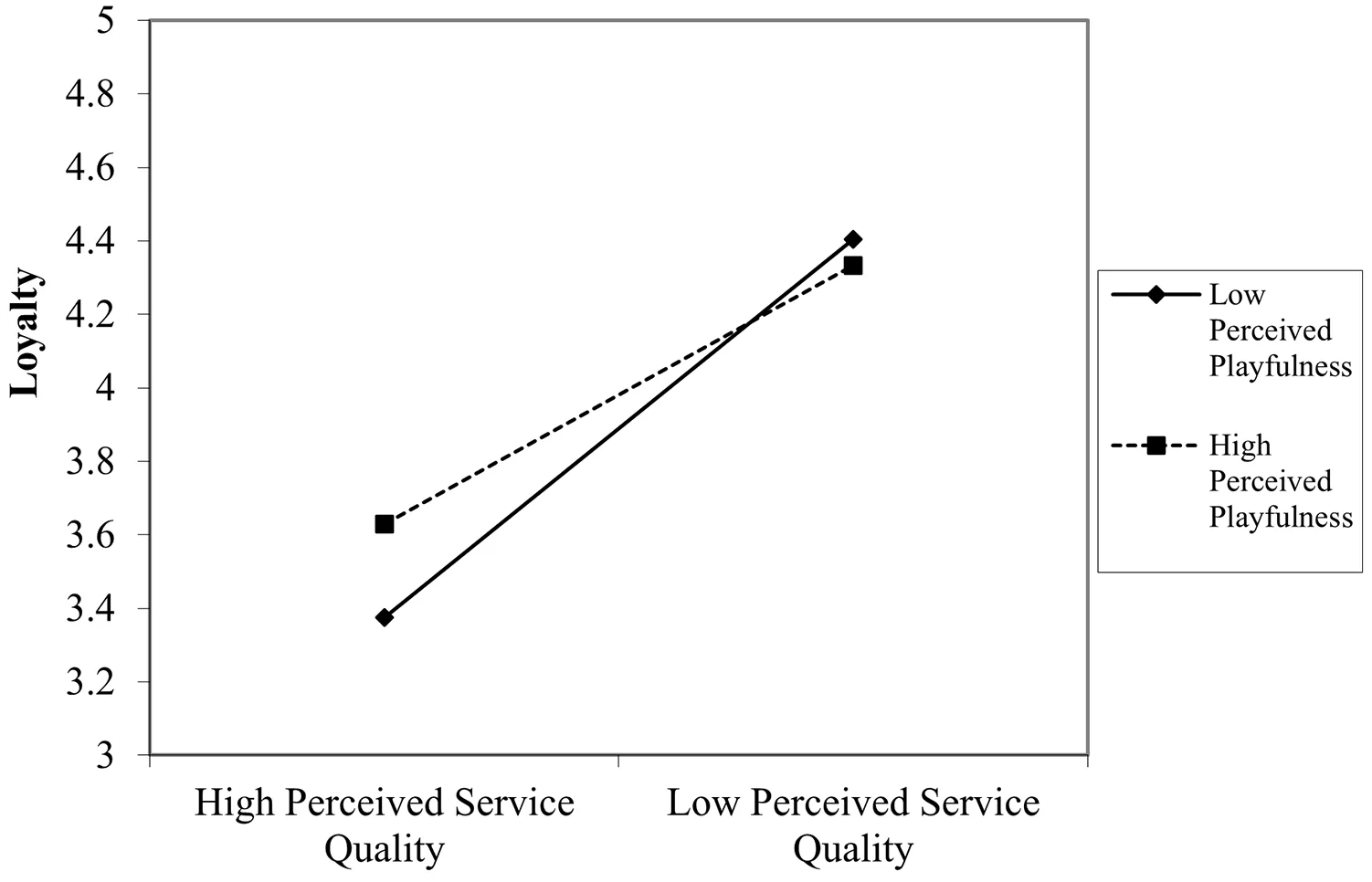
Simple slope analysis of the moderating effect.

### Supplementary analysis

The results of the structural equation model indicated that H6 did not reach statistical significance. Considering the possibility of a more complex association between perceived crowding and loyalty, this study conducted a supplementary analysis to further explore their relationship.

A scatter plot and linear fit curve of the factor scores for perceived crowding and loyalty were generated (Fig 4). The linear fit model yielded an R^2^ of only 0.037, indicating extremely weak explanatory power for the linear relationship. From the perspective of sample distribution, the mean loyalty scores in the low-perceived-crowding and high-perceived-crowding intervals remained at high levels. In contrast, the dispersion of loyalty scores in the medium-perceived-crowding interval was significantly higher than in the extreme intervals. The scatter plot exhibited an inverted triangular distribution (spreading in the middle), suggesting a characteristic of fluctuating heterogeneity. Based on these observations, it is preliminary inferred that the relationship between perceived crowding and customer loyalty is not a simple linear one but may instead involve a potential quadratic non-linear relationship.

**Fig 4:**
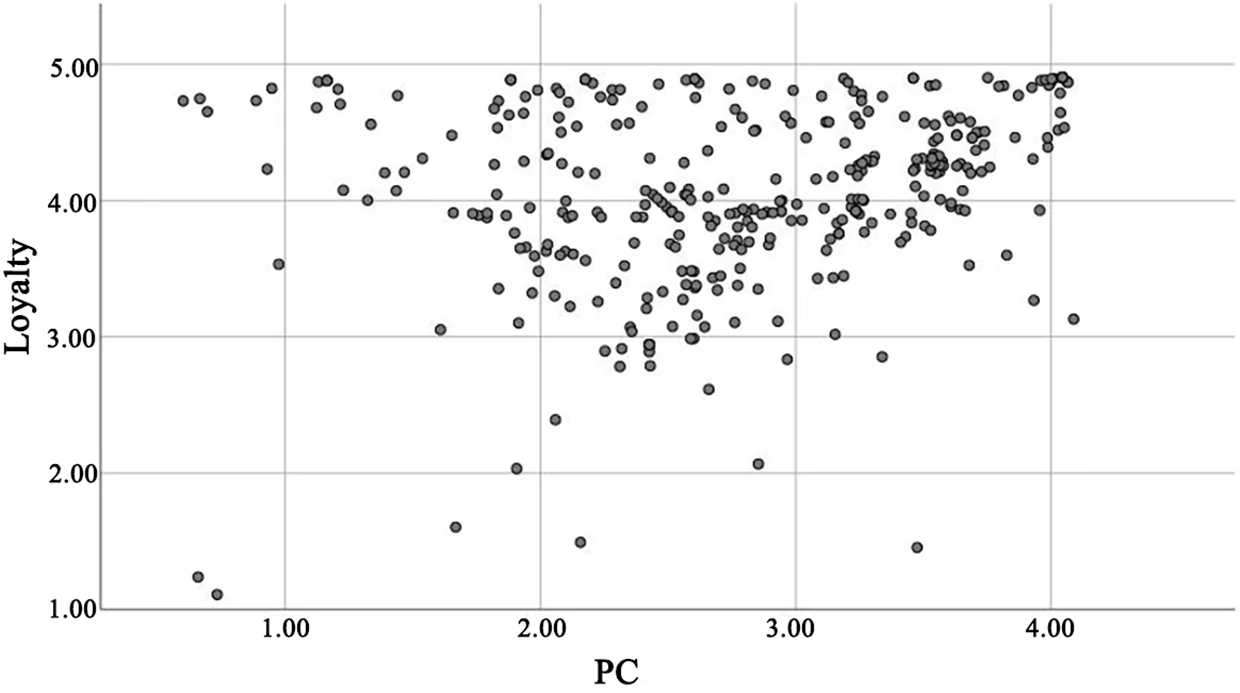
Scatter plot of factor scores for perceived crowding and loyalty.

To further examine the potential non-linear relationship between perceived crowding and loyalty, this study constructed a baseline linear regression model (Model 1) and a quadratic non-linear regression model (Model 2). The estimation results are presented in Table 6.

**Table 6:** Regression results for the relationship between perceived crowding and loyalty.

Variables	Model 1		Model 2	
	β	SD	β	SD
Constant	3.497***	0.126	4.878***	0.292
PC	0.170***	0.045	−1.036***	0.236
PC^2^			0.239***	0.046
R^2^	0.037		0.102	
F	14.328***		21.205***	

The analysis results indicate that compared to the baseline linear model, the non-linear model—which incorporates the quadratic term of perceived crowding—demonstrated a significant improvement in goodness-of-fit, with R^2^ increasing from 0.037 to 0.102, substantially enhancing the model’s explanatory power.

According to the regression coefficients of the quadratic model, the linear term of perceived crowding was significantly negative (β_1_ = −1.036, p < 0.001), while the quadratic term was significantly positive (β_2_ = 0.239, p < 0.001). The calculated turning point (X*) was 2.17, which falls within the measured range of perceived crowding (95% confidence interval: [1.85, 2.49]). Furthermore, the slopes at both ends of the interval were significantly negative and positive, respectively, satisfying the requirements for a U-shaped relationship. These findings confirm a significant U-shaped non-linear relationship between perceived crowding and customer loyalty.

## Discussion

### Discussion of findings

This study finds that even in tourism scenarios driven by social media traffic, the core service quality of tourism products remains a direct, robust, and explanatory predictor of tourist loyalty, effectively replicating and extending the classic performance model of Cronin & Taylor (1992) [21] and supporting its universal applicability across diverse destination types.

While viral online content attracts visitors, the social gains obtained during the visit do not directly trigger loyalty, which instead relies on the establishment of perceived high service quality. This aligns with existing research indicating the indirect role of social value [86]. Furthermore, the positive link between social value and service quality can be explained by self-congruity theory (Sirgy et al., 2000) [87]; when a destination’s image (e.g., “culinary delights” or “vibrant atmosphere” in Tianshui) aligns with a tourist’s self-image, they tend to evaluate local services and functions more favorably. As Hennig-Thurau et al. (2002) further demonstrate, social gains strengthen emotional bonds with the destination and foster psychological bias: when tourists perceive sufficient social benefits, they exhibit greater tolerance for service flaws, strengthening their sense of identity with the destination and ultimately enhancing their positive evaluation of service quality [88].

In the context of “Internet-famous” destinations, perceived crowding exhibits a dual effect: on the one hand, perceived crowding is negatively associated with perceived service quality. This conclusion aligns with the meta-analysis results of Luo et al. (2025), which suggest that at the functional level, cognitive overload triggered by crowded environments generates emotional stress, thereby weakening tourists’ subjective evaluations of the destination’s service quality [89]. On the other hand, perceived crowding at “Internet-famous” destinations is positively associated with perceived social value. A possible explanation is that the high social prestige of the destination satisfies tourists’ bandwagon effect and social comparison needs [90]; the atmosphere of group travel reinforces tourists’ recognition of the rationality of their own travel decisions and confirms the role of the destination’s prestige in enhancing social gains, ultimately exerting a positive impact on perceived social value. This result indicates that high-density crowds are internalized as a key representation of the “Internet-famous atmosphere,” consistent with the view of Jin (2025) that crowding at such destinations has become shareable “social currency” and constitutes a significant dimension of their unique tourism attractiveness [48].

Using a quadratic regression model for testing, it was found that perceived crowding and loyalty exhibit a U-shaped non-linear relationship.. This contrasts with the inverted U-shaped curve between perceived crowding and tourism experience found by Das et al. (2026) in religious tourism [90]. Based on psychological carrying capacity theory [91], the potential explanatory mechanism is that differences in tourists’ psychological carrying capacities serve as the core driver of this U-shaped relationship. Specifically, tourists with high psychological carrying capacity view crowding as an inherent characteristic of “Internet-famous” destinations; they recognize and accept the atmosphere created by the crowds, resulting in minimal interference and a smooth experience, thus maintaining loyalty at a high level. For tourists with low psychological carrying capacity who are sensitive to crowding, although they visit driven by the bandwagon effect and “fear of missing out” (FOMO), the crowding significantly interferes with their visit, leading to psychological stress and avoidance tendencies. However, such tourists may still have high recognition of the destination’s core attractions. Under these dual forces, they may develop a compensatory revisit intention—a desire to return at a different time to complete an “incomplete experience” [91]—which likewise keeps their loyalty at a high level. For tourists with medium psychological carrying capacity, the perceived pressure of crowding is also at a moderate level. At this point, the crowding may disrupt their pace and experience quality, exceeding their comfort threshold without reaching the extreme pressure required to trigger a compensatory motive, thus leading them into a “moderate experience trap.” These tourists neither gain sufficient satisfaction nor develop a strong compensatory revisit intention, resulting in relatively lower loyalty. Notably, although the quadratic model improved R^2^ from 0.037 to 0.102, demonstrating stronger explanatory power, the overall low level of explanation suggests that the mechanism between perceived crowding and loyalty remains complex. Observations of the scatter plot reveal that some tourists’ loyalty remains high regardless of perceived crowding. Therefore, future research should further deconstruct the effects of other mechanisms, such as individual traits and place attachment, on this relationship.

Moderation effect testing indicates that the perceived playfulness of online information negatively moderates the relationship between perceived service quality and loyalty, demonstrating a clear characteristic of hedonic decay in online playful content. This confirms the research findings of Hammedi et al. (2024) on online user behavior, which suggest that the high hedonic value of online information can lead users to shorten their subsequent interest in a product [92]. This study demonstrates that this pattern also exists within offline tourism experiences; while high-hedonic online playful information can attract tourist visits, the repeated stimulation of such playful content simultaneously reduces tourists’ subsequent interest. These results suggest that although online playful marketing regarding a destination is conducive to attracting tourists, it may also impact the destination’s long-term tourist traffic, thereby hindering its sustainable development.

### Limitations

**Temporal limitations of data collection.** The data for this study were collected exclusively during the Qingming Festival holiday from April 4–6, 2024. This period represented a tourism peak driven by the viral spread of online content; consequently, the data primarily reflect tourist experiences under peak-load conditions, which may differ significantly from off-peak periods. This limitation restricts the applicability of the research findings to other timeframes, and the cross-temporal generalizability of the conclusions remains to be further validated in future studies.

**Methodological limitations in sampling.** This study utilized convenience sampling for data collection, which presents inherent limitations regarding the representativeness of the tourist population. Specifically, the sample exhibits a tendency toward homogeneity, predominantly consisting of “highly educated young people.” While this demographic profile aligns with previous research on similar viral cities like Zibo [93], and—as noted by Jager et al. (2017)—homogeneous samples can enhance internal validity by minimizing interference from sub-group extraneous variables [94], it nevertheless restricts the generalizability of the findings to the broader tourism market. Furthermore, data were collected at fixed locations within major scenic spots in Tianshui, which may introduce spatial bias. Finally, due to constraints in manuscript length and sample size, this study did not perform multi-group analysis, potentially overlooking significant variations in research outcomes across different sub-groups.

**Imbalance in sample distribution.** The use of convenience sampling led to imbalances in the sample distribution regarding age and gender. Such imbalances may result in variations in model estimation results across different sub-groups. Due to constraints in length and sample size, this study did not conduct multi-group analysis, which may have resulted in the omission of significant differences between sub-groups.

**Limitations of the moderating variable.** This study utilized “perceived playfulness” as a single-indicator moderating variable. While this reflects tourists’ overall perception of the playfulness of online information, perceived playfulness is a complex emotional construct that may encompass multiple dimensions such as humor, novelty, and social interaction. Consequently, compared to multi-indicator constructs, a single indicator may be less effective in capturing deep-seated psychological structures. Future research could attempt to develop multi-dimensional playfulness scales to further verify the robustness of the conclusions presented in this study.

## Conclusion and Future Research

The findings of this study demonstrate that in the context of “Internet-famous” destinations, perceived service quality is directly and positively correlated with tourist loyalty. Perceived social value is positively associated with loyalty solely through the full mediating effect of perceived service quality. Perceived crowding exhibits a dual impact: it is negatively correlated with perceived service quality but positively correlated with perceived social value; furthermore, it maintains a “U-shaped” non-linear relationship with loyalty. Finally, the perceived playfulness of online information negatively moderates the relationship between perceived service quality and loyalty.

First, subsequent research should aim to overcome specific temporal and spatial constraints. This could involve adopting a longitudinal design to track the evolution of tourist loyalty over time, as well as utilizing probability sampling to expand the coverage area. Such approaches would enhance the external validity and generalizability of the findings, or allow for specialized research and data collection focused on specific sub-groups (e.g., gender-based, youth, or elderly demographics). Second, future studies should include potential tourists. The sample in this study only covered tourists who actually visited the destination, excluding those who possessed a genuine intention to visit but were unable to do so due to objective constraints. This unfulfilled travel intention may exert a “long-tail effect” on future tourist flows and warrants a more in-depth exploration in subsequent research.

## Supporting information

S1 File Raw images. (PDF)
