# Supplementary material for: Structural equation modeling of tourist loyalty in Tianshui, China: Perceived service quality, perceived social value, and crowding effects
Source: PLoS One. 2026 Oct 7;21(10):e0357597. doi: 10.1371/journal.pone.0357597 (PMC13645047; doi:10.1371/journal.pone.0357597)
Supplement: Supplementary file 1 [file pone.0357597.s001.pdf]

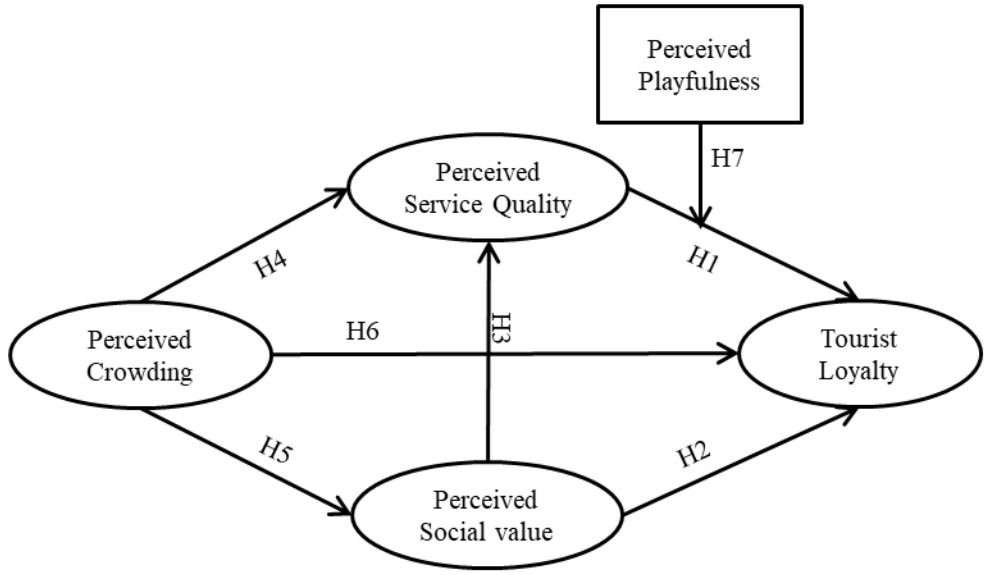
Perceived Playfulness
H7
Perceived Service Quality
H4
H1
Perceived Crowding
H6
H3
Tourist Loyalty
H5
H2
Perceived Social value

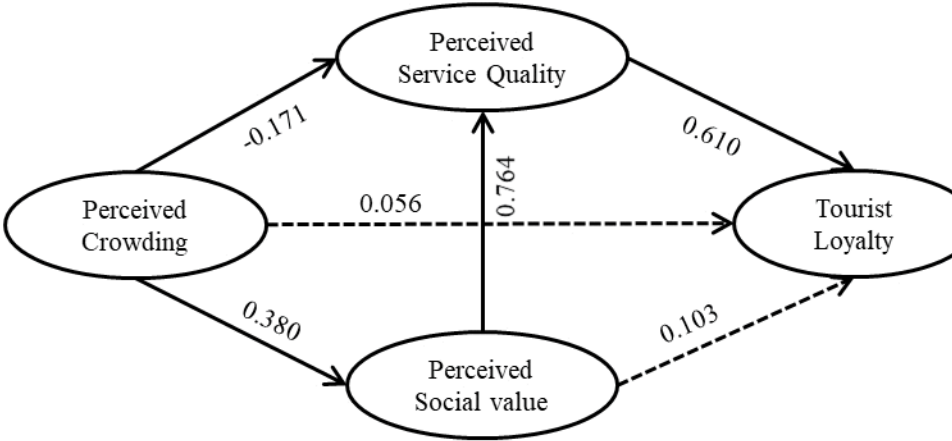

$\chi^2/df = 0.1444$， CFI =0.988， RMSEA = 0.034， SRMR =0.0295， AIC =129.333， TLI = 0.984， GFI = 0.971

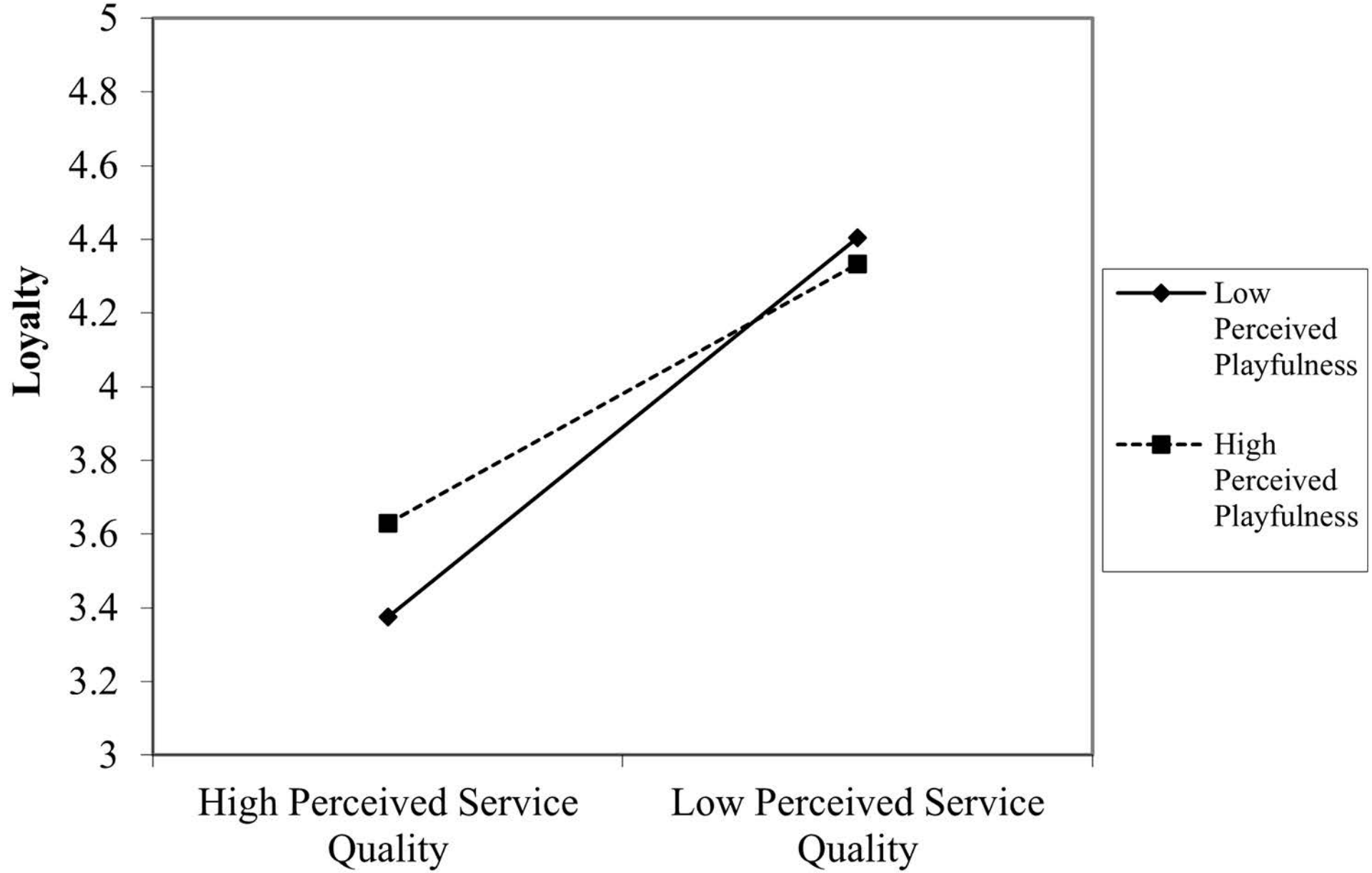

Loyalty
5
4.8
4.6
4.4
4.2
4
3.8
3.6
3.4
3.2
3
High Perceived Service Quality
Low Perceived Service Quality
Low Perceived Playfulness
High Perceived Playfulness

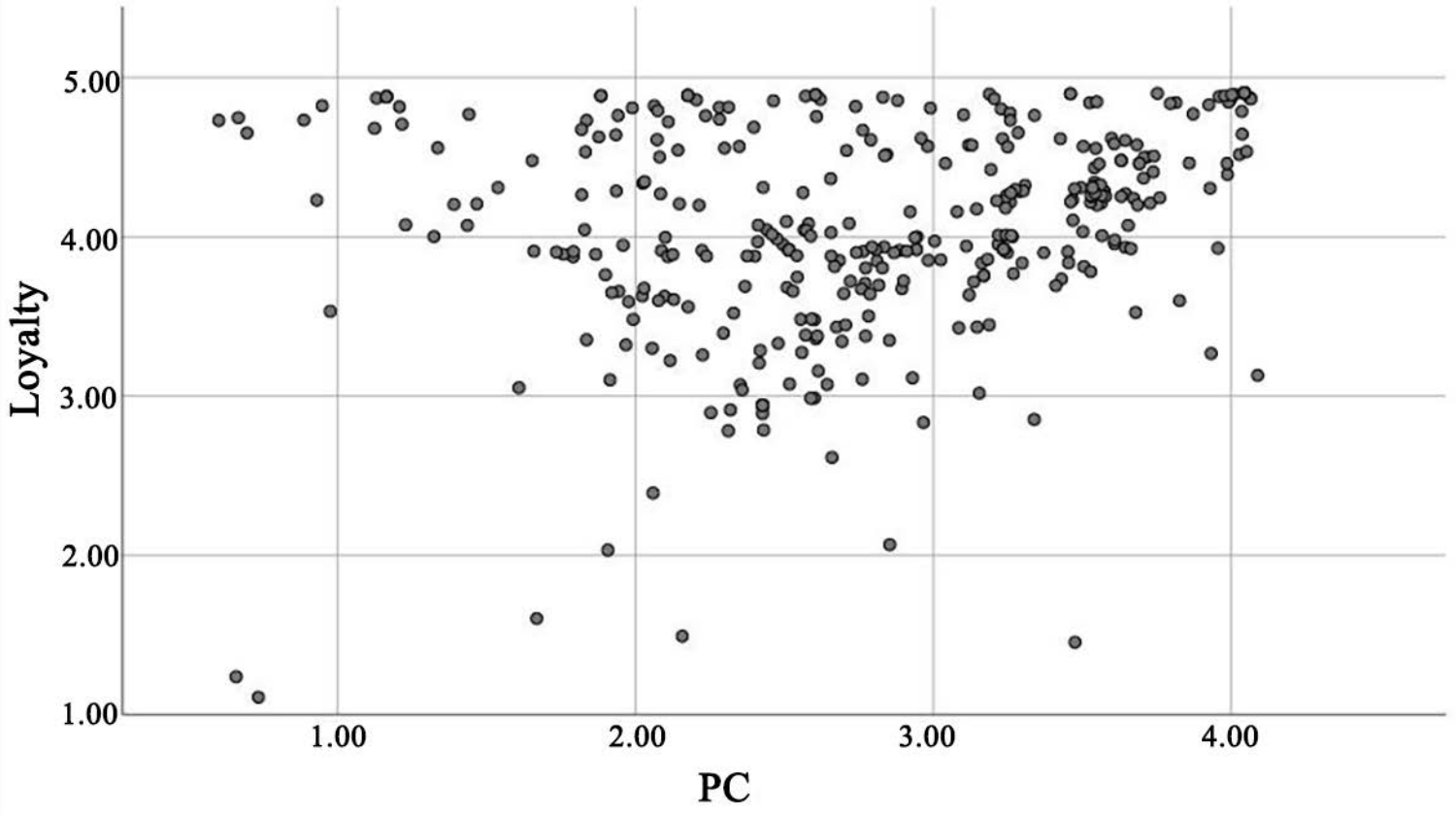
5.00
4.00
3.00
2.00
1.00
Loyalty
1.00
2.00
3.00
4.00
PC
