# Supplementary material for: Structural equation modeling of tourist loyalty in Tianshui, China: Perceived service quality, perceived social value, and crowding effects
Source: PLoS One. 2026 Oct 7;21(10):e0357597. doi: 10.1371/journal.pone.0357597 (PMC13645047; doi:10.1371/journal.pone.0357597)
Supplement: Supplementary file 2 [file pone.0357597.s002.pdf]

Review of Manuscript titled:

Can Becoming an Internet-Famous Destination Influence Tourist Loyalty? An Investigation of Culinary Tourists in Tianshui, China

Abstract

- Aim of the paper as captured in the abstract is completely different from the main titled
- The abstract did not contain any well-defined method
- The results presented are measuring associations without any proved of a known model fitted with the data captured in the abstract
- The conclusion or recommendations appeared as implications of a study which typically deviate from the traditional concept of abstract

Introduction

- The introduction is generally okay
- But from **68-70**, the main topic is different from the sentence such as "This paper seeks to investigate how varying levels of social media interaction contribute to differences in tourist loyalty, aiming to provide additional insights for social media marketing strategies in tourism destinations"

Literature Review and Research Hypotheses

- The two concepts cannot be discussed together as captured

Research context

- I find it difficult to link why this section is placed where it is

Methodology

- The section is poorly written

- There are no sub headings explaining each concept and if possible justification for each

Results

- Table 1, the use of "**gender"** is not appropriate as it explains the binary classification of the biological function of the respondents. Sex will be appropriate
- In Table 1, the classification of **51-60 years** and **60 years and over** should be checked
- The use of descriptive analysis in **222** as a sub heading is not appropriate since Table 1 is also descriptive in nature
- Table 2 position with the narrations should be checked.
- In Table 2 Measurement Reliability and Convergent Validity of the Scale, the emphasis on the **Cronbach's α** is not results but validation of the constructs to assess the internal consistency. Such is often placed in method section
- Chech where Table 3 is placed and the narration

Hypothesis testing

- The nature of reporting this section is not clear

Discussion

- The entire section is done without a single reference to support the findings or disagree with the findings
- Candidate should revise to include references from the literature supporting the work or disagreeing with the study findings

Conclusion and Future Research

- The conclusion should be relooked at
- Including limitations in conclusion is not appropriate

References

- The references should be formatted well

General comments

- Full stops on sub headings should be removed. Check 92, 110, 126 etc
- More work needs to be done on the manuscript
