# Supplementary material for: Structural equation modeling of tourist loyalty in Tianshui, China: Perceived service quality, perceived social value, and crowding effects
Source: PLoS One. 2026 Oct 7;21(10):e0357597. doi: 10.1371/journal.pone.0357597 (PMC13645047; doi:10.1371/journal.pone.0357597)
Supplement: Supplementary file 3 [file pone.0357597.s004.pdf]

**REVIEW MANUSCRIPT DRAFT**

Study on Tourist Loyalty at Internet-Famous Destinations Based on Structural Equation Modeling: Perceived Service Quality, Perceived Social Value, and Crowding Effects

**Suggested title**: Structural equation modeling of tourist loyalty in Tianshui, China: Perceived service quality, perceived social value, and crowding effects

**ABSTRACT**

- In line 24, delete (SEM)
- Use of "we" in line 24 should be delete
- There is no well defined method explained
- There is lack of stated findings
- This is not a discussion session to be drawing inferences

**Introduction**

- Provide reference to support the statement from line 39 to line 42
- From line 100 to line 106, no reference

**Research Hypotheses**

- I sincerely do not see the need to have a preamble which includes line 162 to line 174
- Again, from line 1774 to line 184
- Also, from line 187 to line 193
- From line 196 to line 209 is redundant
- From line 214 to line 221 is also not necessary
- From line 224 to 244 delete
- Having up to seven (7) hypothesis in one study is not an ideal thing to do. Kindly reduce the hypothesis to just two or at most three
- Again, the stated hypothesis, there are null or alternatives

**Materials and methods**

**Data collection**

- In line 274, the use of interviews is not appropriate with the study
- In line 279, the use of 375 valid questionnaires were recovered meant what?
- Provide a reference to support the range of value for the use of the Cronbach's alpha coefficients. You may refer to Taber's interpretations

**Results**

- In line 360, the use of Descriptive statistical analysis should be deleted
- In line 441, Table 2 should be moved to materials and methods if indeed it should be shown
- In Table 3, provide a legend for the acronyms used

**Discussion**

- In line 551, the discussion of findings should be deleted

**Conclusion and Future Research**

- In line 666, the heading should be deleted

**General Comments**

- There is significant improvement in the manuscript
- Authors may have a look at my humble suggestions to improve the content of the manuscript
