# Supplementary material for: Structural equation modeling of tourist loyalty in Tianshui, China: Perceived service quality, perceived social value, and crowding effects
Source: PLoS One. 2026 Oct 7;21(10):e0357597. doi: 10.1371/journal.pone.0357597 (PMC13645047; doi:10.1371/journal.pone.0357597)
Supplement: Supplementary file 4 [file pone.0357597.s005.docx]

# **Response to Reviewers**

## ****1.**** ****Reviewer #1****

### Reviewers' comments:

Abstract

• Aim of the paper as captured in the abstract is completely different from the main titled

• The abstract did not contain any well-defined method

• The results presented are measuring associations without any proved of a known model fitted with the data captured in the abstract

• The conclusion or recommendations appeared as implications of a study which typically deviate from the traditional concept of abstract

### Response

The title has been revised to **"A Study of Tourist Loyalty at 'Internet-Famous' Destinations Based on Structural Equation Modeling: The Roles of Perceived Service Quality, Perceived Social Value, and Crowding Effects."** This updated title more accurately reflects the research focus and the methodological framework.

**Abstract Restructuring:** The abstract has been rewritten to emphasize the application of Structural Equation Modeling (SEM). Furthermore, research inferences have been integrated with empirical measurement results to provide a more cohesive and comprehensive summary of the findings.

**Correction of Causal Terminology:** In alignment with scientific rigor for cross-sectional studies, all causal expressions (e.g., "influences," "leads to") have been replaced with relational or associational terminology (e.g., "is associated with," "is correlated with," or "predicts") to accurately reflect the links identified in the model.

**Refinement of Expression:** The overall phrasing and logical flow have been updated to ensure that the theoretical deductions are strictly supported by the measurement outcomes, thereby enhancing the clarity and consistency of the manuscript.

### Reviewers' comments:

Introduction

• The introduction is generally okay

• But from 68-70, the main topic is different from the sentence such as “This paper seeks to investigate how varying levels of social media interaction contribute to differences in tourist loyalty, aiming to provide additional insights for social media marketing strategies in tourism destinations”

### Response

**Optimization of the Introduction:** The introduction has been thoroughly restructured and optimized to establish a stronger logical connection with the revised title. Consequently, the previously mentioned text has been removed to ensure a more coherent and focused opening that better sets the stage for the research objectives.

### Reviewers' comments:

Literature Review and Research Hypotheses

• The two concepts cannot be discussed together as captured

Research context

• I find it difficult to link why this section is placed where it is

Methodology

• The section is poorly written

• There are no sub headings explaining each concept and if possible justification for each

### Response

**Section Decoupling:** The original section titled "Literature Review and Research Hypotheses" has been decoupled into two independent sections: **"1. Research Framework"** and **"2. Research Hypotheses."** This restructuring aims to provide a clearer distinction between the overarching theoretical logic and the specific path assumptions, thereby improving the manuscript's navigational clarity.

**Removal of Research Background:** The "Research Background" section has been removed to maintain a more concise and focused introduction.

**Expansion of "Materials and Methods":** The original "Materials and Methods" section has been expanded and restructured into three distinct subsections: **"Study Area,"** **"Data Collection,"** and **"Variable Measurement."** This restructuring provides a more granular and systematic description of the research methodology.

**Optimization of Phrasing:** The manuscript's overall phrasing has been refined for improved clarity and academic precision.

**Expansion of "Variable Measurement":** A dedicated "Variable Measurement" section has been added. In this section, we provide detailed explications of the **conceptual connotations** and the **theoretical rationale** for all variables used, ensuring that each construct is both well-defined and robustly justified within the research context.

### Reviewers' comments:

Results

• Table 1, the use of “gender” is not appropriate as it explains the binary classification of the biological function of the respondents. Sex will be appropriate

• In Table 1, the classification of 51-60 years and 60 years and over should be checked

• The use of descriptive analysis in 222 as a sub heading is not appropriate since Table 1 is also descriptive in nature

• Table 2 position with the narrations should be checked.

• In Table 2 Measurement Reliability and Convergent Validity of the Scale, the emphasis on the Cronbach's α is not results but validation of the constructs to assess the internal consistency. Such is often placed in method section

• Chech where Table 3 is placed and the narration

### Response

**Refinement of Demographic Categories:** In Table 1, the variable "Gender" has been replaced with **"Sex"** for biological accuracy. Additionally, the age category "60 and above" has been updated to **"61 and above"** to ensure precise, non-overlapping classification.

**Subsection Reordering:** The subheading **"Descriptive Analysis"** has been relocated to precede Table 1. The overall content of this section has been restructured to enhance the logical progression of the results.

**Sequential Optimization of Tables:** * **Table 2** (Measurement Model results) has been repositioned immediately following the **full-sample Confirmatory Factor Analysis (CFA)** section.**Table 3** has been placed following Table 2 to maintain a systematic presentation of the empirical evidence.

**Methodological Enhancement:** In the "Variable Measurement" subsection of the Methods, the results regarding **Cronbach’s alpha** coefficients have been integrated to provide early verification of the scales' reliability.

### Reviewers' comments:

Hypothesis testing

• The nature of reporting this section is not clear

Discussion

• The entire section is done without a single reference to support the findings or disagree with the findings

• Candidate should revise to include references from the literature supporting the work or disagreeing with the study findings

Conclusion and Future Research

• The conclusion should be relooked at

• Including limitations in conclusion is not appropriate

### Response

**Restructuring of the Results Section:** The Results section has been expanded into four distinct subsections to provide a more systematic presentation of the empirical findings: **Path Analysis**, **Mediation Effect Testing**, **Moderation Effect Testing**, and **Supplementary Analysis**.

**Comprehensive Rewrite of the Discussion:** The Discussion section has been entirely rewritten. We have incorporated extensive supporting evidence from the existing literature to contextualize our findings and provided more in-depth theoretical explanations for the observed relationships.

**Update of Concluding Remarks:** The **Conclusions**, **Limitations**, and **Future Research** sections have been rewritten to reflect the refined analytical results and to provide more nuanced insights for both theory and practice.

### Reviewers' comments:

References

• The references should be formatted well

General comments

• Full stops on sub headings should be removed. Check 92, 110, 126 etc

• More work needs to be done on the manuscript

### Response

**Standardization of References:** All references have been formatted strictly according to the journal's specific requirements.

**Correction of Heading Formatting:** The periods (full stops) following all subheadings have been removed .

**Comprehensive Manuscript Review:** A thorough, top-to-bottom revision of the entire manuscript has been conducted to ensure linguistic consistency, logical coherence, and formatting precision.

## Reviewer #2:

### Reviewers' comments:

1. Theoretical framing and hypotheses
Conceptualisation of “perceived social value” is narrow and overlaps with other constructs.
The scale items capture social attention, popularity, making friends, and self-confidence (X1–X4), which mix social status, social relationship gains, and self-related benefits under one factor, yet the theory section does not justify why these heterogeneous aspects belong to a single latent construct or how it differs from social image/value, self-enhancement, or hedonic value.
Operationalisation does not fully reflect SOR and UGT arguments.

### Response

We reconstructed the **"Perceived Social Value"** construct based on the social value dimension of the PERVAL scale developed by **Sweeney and Soutar (2001)**. Three indicators were retained: "This trip increases my social media followers," "This trip makes me more popular among friends," and "This trip helps me make more friends." The indicator "This trip makes me more independent and confident" was removed due to its conceptual ambiguity with social value. All indicators were renumbered accordingly, and the revised construct successfully passed the model validation.

Sweeney J C, Soutar G N. Consumer perceived value: The development of a multiple item scale. Journal of retailing, 2001, 77(2): 203-220.

### Reviewers' comments:

Operationalisation does not fully reflect SOR and UGT arguments.

The SOR framing suggests multiple types of stimuli (UGC, authenticity of viral atmosphere, congruence between online and offline experience), but the model only includes perceived crowding and perceived social value as “stimuli”, with experience quality as “organism” and loyalty as “response”; key digital elements (e.g., online authenticity, congruence, expectations) are discussed conceptually but not measured.

### Response

We have re-evaluated the theoretical framework of this study. The content related to **Stimulus-Organism-Response (SOR)** and **Uses and Gratifications Theory (UGT)** has been removed. Instead, we have restructured the research framework to focus more precisely on the interplay between **perceived service quality** and **perceived social value**, ensuring a more cohesive and streamlined logical flow.

### Reviewers' comments:

H1 is not logically integrated with later mediation.
The hypothesis states that perceived social value directly increases loyalty (H1), then the mediation section argues that experience quality fully mediates this relationship and that social value has no direct effect on loyalty. This is not just a statistical outcome; it contradicts the initial claim that social value is a direct antecedent of loyalty, and there is no ex-ante rationale for why the effect should become fully indirect.

### Response

Based on our literature review, existing research indicates that perceived social value is a precursor to loyalty, primarily exerting an indirect influence (Preko A., et al., 2020; Kim M., et al., 2018). Accordingly, the revised "Research Hypotheses" section clarifies this relationship and adopts a testing procedure that examines direct effects before proceeding to mediation analysis.

Preko A, Mohammed I, Ameyibor L E K. Muslim tourist religiosity, perceived values, satisfaction, and loyalty. Tourism Review International, 2020, 24(2-3): 109-125.

Kim M, Thapa B. Perceived value and flow experience: Application in a nature-based tourism context. Journal of destination marketing & management, 2018, 8: 373-384.

### Reviewers' comments:

Contradiction in the treatment of H6.

H6 hypothesises “no significant influence” of perceived crowding on loyalty. The structural model finds a non-significant path (β = 0.044, p > 0.05) and labels H6 as “Accept.”

Accepting a null hypothesis is statistically contentious; usually one “fails to reject” it and would need power analysis or equivalence testing to support a claim of “no effect”. This should be reformulated or justified more rigorously.

### Response

**Refined Analytical Approach and Theoretical Integration:** Following your constructive feedback, we have revised our testing methodology. Drawing on **Stress Overload Theory**, we initially hypothesized a negative impact of perceived crowding on tourist loyalty. Upon finding the linear hypothesis unsupported, we conducted a supplementary analysis to explore their underlying relationship. A quadratic regression model revealed a **U-shaped non-linear relationship**. We have interpreted this phenomenon through the lenses of **Psychological Carrying Capacity**, the **Bandwagon Effect**, and **Fear of Missing Out (FOMO)**. These updates have been integrated into the "Research Hypotheses," "Hypothesis Testing," and "Discussion of Findings" sections.

### Reviewers' comments:

Moderation by “perceived comment interaction” is under-theorised and minimally measured.

The moderation hypothesis (H7) is based on Uses-and-Gratifications Theory, but the moderator is measured by a single item (“I think the comment section … is very interesting”), which mainly reflects entertainment rather than interaction or participation (no frequency, depth, or type of engagement).

This weak alignment between theory (active co‑creation, gratifications) and measurement (interest in comments) raises construct validity concerns.

### Response

We have repurposed the item "I find the comment section very interesting" to measure **Perceived Playfulness**. As hubs for secondary creation and **Internet Memes**, comment sections offer humor and social resonance that drive sensory satisfaction.

Furthermore, we have adopted **Hedonic Declension Theory** to explain the negative moderating effect of perceived playfulness on the relationship between service quality and loyalty. According to Wathieu (2004), consumers internalize past experiences as "reference points," requiring higher intensity or novelty to maintain satisfaction. The psychological response to repeated stimuli evolves through **sensitization, habituation, and satiation**. Galak (2018) and Chen (2019) suggest that repeated exposure generates negative cumulative effects; even satisfied customers may develop negative attitudes toward future consumption if they anticipate declining marginal utility.

In this study, we argue that while geographical distance once limited repetitive stimuli, short-video algorithms now enable high-frequency exposure to destination content. This compresses the cycle from sensitization to **satiation**. Pre-experiencing "viral" playful content leads to an early onset of hedonic declension, thereby weakening the formation of long-term loyalty and manifesting as a negative moderating effect.

Wathieu, L. Consumer habituation. Management Science, 2004,50(5), 587-596.

Becerril-Castrillejo, I., & Munoz-Gallego, P. A. Influence of habitual level of consumption on willingness to pay: A satiation, sensitization, and habituation approach. International Journal of Hospitality Management,2022, 103, 103210.

Galak, J., & Redden, J. P. The properties and antecedents of hedonic decline. Annual review of psychology, 2018, 69, 1-25.

Chen, C. C., & Liao, Y. S. From satisfaction to satiation: The gradation model of satiation in casual leisure. Annals of Leisure Research, 2019, 22(2), 233-246.

### Reviewers' comments:

2. Research design and external validity

Single destination and very narrow time window.

Data are collected only in Tianshui during one holiday (Qing-Ming, 4–6 April 2024), within a specific boom period. This severely limits generalisability to other “internet-famous” destinations, off‑peak periods, or other types of viral events. The paper calls the setting a “quasi-experimental window” but does not employ any quasi-experimental design (no pre‑post, control group, or natural experiment structure).

### Response

We fully acknowledge that data collected during a single holiday primarily reflects tourist experiences under peak conditions, which may limit the generalizability of the findings to off-peak periods. This limitation is now explicitly discussed in the "Research Limitations" section.

However, we contend that data captured during peak periods is uniquely suited to reflect tourist perceptions during **viral tourism events**. Since 2023, China has witnessed a surge in tourism phenomena characterized by **short-video-driven, large-scale influxes of tourists in extremely short periods**. This "online traffic-driven + offline surge" development model has emerged as a significant new pattern in Chinese tourism (Ye et al., 2026), warranting focused academic inquiry.

The data collection during the Qingming Festival (April 4–6) occurred at the peak of the destination’s popularity. Conclusions derived from this relatively extreme environment offer valuable insights into understanding the tourism booms triggered by viral marketing. Additionally, we agree that the term "quasi-experimental window" is inappropriate; we have removed this phrasing and clarified that this study is primarily a **research case study**.

Ye, C., & Zhang, Y. Traffic "Carnival" or "Frenzy": The Tourism Paradox in the Digital Age[J/OL]. *Geographical Science*,2026, 1-10. <https://doi.org/10.13249/j.cnki.sgs.20260100>.

### Reviewers' comments:

Sample composition may bias results but is not critically discussed.

The majority are 18–25 years old (58.7%), low-income, and highly educated (72% bachelor/college diploma), which is not representative of the broader tourist market. Given that social media use and comment interaction are age- and education-sensitive, the findings may primarily describe young social-media-savvy tourists; this limitation is not sufficiently emphasised.

### Response

We fully concur with your assessment and have addressed this issue in the "Research Limitations" section. Our study utilized **convenience sampling**, which inherently limits the representativeness of the tourist population. This resulted in a **homogeneous sample**, predominantly composed of "highly educated young people."

While this demographic aligns with recent findings on similar "Internet-famous" cities like Zibo (Yang et al., 2024), and such homogeneity can enhance **internal validity** by minimizing confounding sub-group variables (Jager et al., 2017), we acknowledge that it constrains the generalizability of our findings to the broader tourism market. Furthermore, the localized data collection at Tianshui’s major attractions may have introduced **spatial bias**. In the "Future Research" section, we now propose that subsequent studies should employ **probability sampling** and broader coverage to strengthen **external validity** and universal applicability.

Yang S, Liu Y, Xu L. The effect of food tourism experiences on tourists’ subjective well-being. Heliyon, 2024,10 (3), e25482[EB/OL].

Jager, J., Putnick, D. L., & Bornstein, M. H. II. More than just convenient: The scientific merits of homogeneous convenience samples. Monographs of the society for research in child development, 2017, 82(2), 13-30.

### Reviewers' comments:

Potential common method bias is not addressed.

All constructs (perceived social value, experience quality, crowding, loyalty, and the moderator) are measured via self-report, in the same questionnaire, at a single time point; yet there is no mention of Harman’s one-factor test, CFA marker-variable tests, or other checks for common method variance.

### Response

To ensure the rigor of our empirical results, we conducted a Common Method Bias test. The procedures and results have been detailed in the "Measurement Model Testing" section:

A Harman’s single-factor test was conducted as a preliminary assessment. The unrotated exploratory factor analysis (EFA) of all scale items revealed that the first principal factor explained 41.593% of the variance. As this slightly exceeded the 40% threshold, it suggested the potential presence of common method variance (CMV). Following the recommendations of Tang et al. (2020), the CFA marker variable technique was employed to further verify the severity of this bias. A marker variable (M), unrelated to the research variables, was added to the CFA model to construct four sequential models: the baseline model (assuming no CMV), Model C (assuming CMV with equal influence on all indicators), Model U (assuming CMV with varying influence on all indicators), and Model R (assuming CMV without affecting correlations between variables).

The chi-square differences between these models were estimated and compared. A significant chi-square difference was observed between the baseline model and Model C ( Δχ² (Δdf = 1) = 7.762,p = 0.005), indicating the presence of CMV. Furthermore, the difference between Model C and Model U was significant (Δχ²(Δdf = 11) =19.703, p = 0.049), showing that CMV influenced the indicators differently. However, no significant difference was found between Model U and Model R (Δχ² ( Δdf = 6 )=0.968, p =0.987). In summary, while slight common method variance exists in the data, it does not constitute a severe common method bias (CMB) and will not substantially interfere with the research results or hypothesis testing. Thus, the data are suitable for subsequent structural model analysis.

Tang D D, Wen Z L. Statistical Approaches for Testing Common Method Bias: Problems and Suggestions. Journal of Psychological Science, 2020, 43(1): 215-223.

### Reviewers' comments:

Cross-sectional data used to infer directional/causal relations.
The study interprets paths as “influences” (e.g., perceived social value → experience quality; experience quality → loyalty), but the design is purely cross-sectional and observational; no temporal separation or experimental manipulation supports causal claims. A Q1 journal would expect a more cautious interpretation.

### Response

We fully agree with your suggestion. Consequently, we have replaced all causal terminology throughout the manuscript with associational language. Specifically, terms such as **"associated with,"** **"related to,"** and **"co-vary"** are now used instead of "cause" or "determine" to more accurately reflect the nature of our statistical findings and avoid overstating the causal links.

### Reviewers' comments:

3. Measurement and model specification

Weakness in perceived crowding measurement.

Perceived crowding is measured with only two items: “congestion impacted my travel experience” and “I should avoid hotspots and come back later” (X10–X11).

These conflate: perceived density (crowding), affective reaction, and behavioural intention (avoidance).

Thus, the construct is not a pure measure of “crowding” and overlaps conceptually with experience quality and loyalty/behavioural intentions, which may distort path estimates.

### Response

We appreciate your insightful comments regarding the conceptual definition of our measurement items. We have updated the "Variable Measurement" section to justify the selection of these indicators.

Based on pre-survey feedback, we adjusted the measurement of Perceived Crowding. In viral tourism contexts, objective crowding is a ubiquitous fact; thus, basic perceptual items (e.g., "It is too crowded here") often exhibit high homogeneity and lack statistical variance. Consequently, we utilized perceived crowding stress as a proxy to capture psychological overload.

Drawing on Cognitive Appraisal Theory (CAT) (Lazarus, 1991), individual responses to stressors encompass both internal emotional experiences and external coping tendencies, which are psychologically coherent (Gross, 2015). We selected two specific items: "Crowding affected my travel experience" (focusing on goal interference and internal loss appraisal) and "I should avoid hotspots and return later" (reflecting stress-driven avoidance and external coping tendencies). Together, these items represent a comprehensive stress response at both cognitive and behavioral levels (Neuts, 2012), providing deeper insights and necessary statistical differentiation. Confirmatory Factor Analysis (CFA) results confirm that this construct meets all requirements for convergent and discriminant validity.

Lazarus R S. Emotion and Adaptation. Oxford University Press; 1991.

Gross J J. Emotion regulation: Current status and future prospects. Annual Review of Psychology. 2015; 66: 1-24.

Neuts B, Nijkamp P. Tourist crowding perception and acceptability in cities: An applied modelling study on Bruges. Annals of Tourism Research. 2012;39(4):2133-2153.

### Reviewers' comments:

Marginal reliability for perceived crowding.
Cronbach’s α and composite reliability for perceived crowding are both 0.699, just below or at the threshold; the text states they “meet or approach” 0.70, but this borderline reliability, combined with only two items, should be more critically discussed.

### Response

Following the perspective of **Hulin & Cudeck (2001)**, raw inter-item correlations often underestimate the true reliability of two-item scales; thus, **Composite Reliability (CR)** based on Spearman-Brown correction logic offers a more accurate reflection of internal consistency. The "Crowding Response" dimension in this study comprises two items, and as such, CR was utilized as the primary reliability metric.

Statistical analysis reveals that the means, standard deviations (SD), and factor loadings of both items are highly consistent, exhibiting characteristics of **essentially parallel measures**. The reconstructed model yielded a CR of **0.701** (exceeding the 0.70 threshold) and an **Average Variance Extracted (AVE) of 0.540** (exceeding the 0.50 threshold), satisfying the requirements for convergent validity. Consequently, both the reliability and validity of this scale align with rigorous academic standards.

Hulin, Charles, and Robert Cudeck. "Cronbach’s alpha on two-item scales." Journal of Consumer Psychology, 2001, 10.1/2: 55.

### Reviewers' comments:

Ad-hoc deletion of X9 may affect construct validity.

Item X9 (“Long queuing time for Malatang negatively affected my tourism experience”) is deleted because of a low factor loading (0.445), improving fit indices. However:

Theoretical implications are not fully examined (queuing is central to viral food destinations; removing it may truncate the experience-quality dimension).

### Response

We appreciate the opportunity to clarify the removal of item X9 ("Long queuing time for Malatang negatively affected my tourism experience"). Our data shows that 84.8% of tourists visited Tianshui primarily for its Malatang, and 81.3% were aware of the crowding beforehand. Drawing on **Stimulus-Overload Theory**, we argue that queuing for Malatang has been internalized as a unique, pre-anticipated component of the "Internet-famous" destination experience rather than a purely negative stressor. The low factor loading of X9 suggests that the perceived stress from queuing did not reach the threshold of **"overload,"** failing to converge significantly with the sensory overload construct.

Removing X9 allowed us to focus more precisely on the actual factors triggering sensory overload, thereby enhancing the theoretical validity of the Perceived Crowding dimension. While the EFA initially suggested potential cross-loadings or factor merging following the reduction of items, we maintained the theoretically grounded four-dimensional structure for CFA to prevent information loss. The **CFA results confirmed robust structural validity**, demonstrating that the item deletion did not compromise the model’s integrity.

### Reviewers' comments:

No cross-validation is performed; the deletion is based on a single split-sample CFA.

The crowding construct then no longer covers queueing, which is a key aspect of “viral” crowding.

Use of split-sample EFA/CFA with moderate N.

With N = 375, the authors split into N = 167 and N = 208 for EFA and CFA. While methodologically neat, this reduces power and stability in both analyses; a Q1 journal might ask for additional validation (e.g., full-sample CFA, or cross-validation with an independent sample).

### Response

To enhance the robustness of our findings, we randomly split the total sample into two equal halves. The first half was used for **Exploratory Factor Analysis (EFA)** to determine the factor structure, while the second half was used for **Confirmatory Factor Analysis (CFA)** for structural validation. Subsequently, a **full-sample CFA** was conducted to verify the final model's stability and reliability. This cross-validation approach ensures that our measurement model is both statistically sound and generalizable.

EFA was conducted on the first subsample (N = 182) using SPSS 26.0. The KMO value was 0.837, and Bartlett’s test of sphericity yielded χ² = 818.374 (p < 0.001), indicating that the data were suitable for factor analysis. Factors were extracted using the maximum likelihood method. With the eigenvalue-greater-than-1 criterion, the cumulative variance explained was 64.685%. However, the rotated component matrix exhibited cross-loadings and dimension merging, extracting only three factors, which was inconsistent with the theoretical framework of this study. Further assessment of the scree plot revealed that the eigenvalue for a four-dimension structure was 0.970, approaching the critical threshold of 1.0. Considering that a strict reliance on eigenvalues may result in the loss of essential structural information, this study retained the preset four-dimension structure. The construct validity was subsequently subjected to further testing through confirmatory factor analysis (CFA).

CFA was performed on the second subsample (N = 193) using Amos 26.0. The results indicated a good model fit ( χ²/df = 1.136, CFI = 0.994, RMSEA = 0.026, SRMR =0.0348, AIC =114.550, TLI = 0.992，GFI = 0.957). All standardized factor loadings exceeded 0.60 ( p < 0.001 ), demonstrating that the measurement model was well-specified. Furthermore, all Average Variance Extracted (AVE) values were greater than 0.50, and the square root of the AVE for each factor was higher than its correlation with any other factor. These results confirm that the four-dimension structure possesses robust construct validity, including both convergent and discriminant validity.

A CFA was further conducted using the full sample (N = 375). The results maintained a high level of model fit ( χ²/df =1.444, CFI =0.988, RMSEA = 0.034, SRMR =0.0295, AIC =129.333, TLI = 0.984, GFI = 0.971) . The standardized factor loadings for all items ranged from 0.634 to 0.834 (p < 0.001). The Composite Reliability (CR) values ranged from 0.701 to 0.856, and the Average Variance Extracted (AVE) for all constructs exceeded 0.50, providing strong support for convergent validity. Furthermore, the square root of the AVE for each factor was greater than its correlation with any other factor, supporting robust discriminant validity.

### Reviewers' comments:

Single-item moderator and lack of measurement model integration.
Perceived comment interaction is treated outside the SEM and estimated via hierarchical regression using factor scores, rather than as a latent construct within the SEM framework. This introduces:

♣ inconsistency between the main SEM and moderation test,

♣ measurement error not accounted for in interaction effects, and

♣ a risk of biased moderation estimates.

### Response

We appreciate your professional advice. Incorporating the moderator as a latent construct within the Structural Equation Modeling (SEM) framework is indeed crucial. Using factor scores in our original hierarchical regression neglected the impact of measurement error on the estimation of moderation effects. To address this, we have implemented the following improvements:

The following improvements were implemented within the **Structural Equation Modeling (SEM)** framework:

Construction of Single-Indicator Latent Variables: Instead of factor scores, we adopted the Fixed Error Variance Method (Hayduk, 1987). Based on a conservative reliability estimate (r = 0.80) and the observed variance (0.928), the residual variance was fixed at 0.1856. This approach explicitly accounts for measurement error, ensuring more accurate parameter estimation.

Interaction Modeling with Residual Centering: To mitigate multicollinearity and enhance interpretability, we employed orthogonalized (residual) centering for the interaction term (Little et al., 2006). The centered interaction and moderator were integrated into the main SEM model for Maximum Likelihood (ML) estimation.

Full-Model Validation and Simple Slope Analysis: The refined model demonstrated a good fit (χ²/df = 3.287, CFI = 0.902, RMSEA = 0.078). The path coefficient for the interaction term remained significant (β = -0.215, p < 0.001), confirming the robustness of the moderating effect. Furthermore, Simple Slope Analysis was conducted to further elucidate the pattern of moderation and validate our hypotheses.

|  | Low Perceived Playfulness | High Perceived Playfulness |
| --- | --- | --- |
| High Perceived Service Quality | 3.37541 | 3.62887 |
| Low Perceived Service Quality | 4.40459 | 4.33313 |

Fig1 . Simple slope analysis of the moderating effect

Hayduk L A. Structural equation modeling with LISREL: Essentials and advances. Jhu Press; 1987.

Little, Todd D., James A. Bovaird, and Keith F. Widaman. "On the merits of orthogonalizing powered and product terms: Implications for modeling interactions among latent variables." Structural equation modeling 13.4 (2006): 497-519.

### Reviewers' comments:

No test of measurement invariance or robustness.

Given the strong age skew and possible gender differences, the study does not test whether the measurement model is invariant across subgroups (e.g., male vs female, younger vs older). For a Q1 outlet, multi-group analysis or at least a discussion of this omission would be expected.

### Response

We agree that sub-group differences are important. However, given the **sample size limitations**, conducting a rigorous **multi-group analysis** was not feasible for this study. We have explicitly added this as a limitation and suggested that future research should employ larger, targeted samples to explore how variables like age and gender may moderate the identified paths.

### Reviewers' comments:

4. Data analysis and interpretation

Inconsistent description of hypothesis support.

The text states: “Paths 2, 3, 4 and 5 are significant, whereas paths 1 and 6 are not. Consequently, the model supports H2, H3, H4, H5 and H6.”

This is logically inconsistent: H6 explicitly hypothesises a non-significant effect, but the “support” is derived from a non-significant coefficient without proper equivalence testing. At minimum, the wording should distinguish “confirmed no significant effect” from “supported effect.”

### Response

Following the **Stress Overload Theory**, we revised H6 to propose a negative linear relationship. However, as the data did not support this, we conducted a **quadratic regression analysis** which uncovered a significant **U-shaped relationship**. We have now restructured the manuscript to discuss how moderate crowding initially deters loyalty, while extreme crowding (at "Internet-famous" sites) may paradoxically reinforce loyalty through the **Bandwagon Effect**.

### Reviewers' comments:

Moderation result is statistically fragile.

The interaction term has β = –0.115 with a standard error of 0.129 according to Table 6, yet it is claimed as significant at 1%. A β smaller in magnitude than its standard error would normally not yield high significance; this suggests a possible typo or reporting error (in β, SE, or p‑value). A Q1 reviewer would ask for recalculation, reporting of t‑value, and perhaps the full regression output.

### Response

We apologize for this **transcription error**. Upon re-checking our original SPSS output, we confirmed that the **Standard Error** values for the interaction and moderator terms were accidentally swapped during manual entry. We have corrected these values in the revised manuscript (Table 1). The original software output is provided below for your verification. We appreciate your meticulous review

| Table 1 **coefficient^a^** | | | | | | |
| --- | --- | --- | --- | --- | --- | --- |
| Model | | Unstandardized coefficient | | standardized coefficient | t | p |
|  |  | B | S.E | Beta |  |  |
| 1 | constant | -.925 | .474 |  | -1.952 | .052 |
|  | PSQ | 1.423 | .149 | 1.055 | 9.540 | .000 |
|  | interaction term | -.115 | .039 | -.764 | -2.948 | .003 |
|  | moderator | .415 | .129 | .633 | 3.210 | .001 |
| a. Dependent variable: Loyalty | | | | | | |

We have corrected the typographical errors in the data: the interaction term yields β = -0.115 (S.E. = 0.039, t = -2.948, p < 0.01). Following your advice, we now prioritize **SEM-based latent interaction modeling** in the main text to confirm the moderation effect. The original regression analysis results have been moved to the **"Robustness Check"** subsection to provide supplementary evidence for our findings.

### Reviewers' comments:

Effect sizes and practical significance are not reported.

R² values are given, but there is no discussion of effect sizes (e.g., standardized indirect effects, variance in loyalty explained uniquely by social value vs crowding) or whether differences are substantively meaningful.

### Response

The moderating effect has been formally tested within the SEM framework, showing a significant negative (dampening) effect that supports our hypotheses. Although the R^2^ decreased slightly from 0.512 to 0.490, we attribute this to the increased model complexity and potential error dilution in complex SEM structures (Kenny, 2018). Given that the interaction path coefficient remains significant and theoretically consistent, the conclusions regarding the moderating effect remain robust.

Kenny, D. A. (2018). Moderator variables: introduction. http://davidakenny.net/cm/moderation.htm.

### Reviewers' comments:

No consideration of alternative models.

The model assumes a specific ordering: social value and crowding → experience quality → loyalty. However, plausible alternatives (e.g., experience quality influencing perceived social value; loyalty influencing intention to avoid hotspots) are not tested or discussed. A Q1 journal often expects at least a brief consideration of competing specifications.

### Response

We appreciate the reviewer’s constructive suggestions. To ensure the statistical robustness of our hypothesized model, we conducted a **competing model analysis** by testing alternative causal directions. We compared the model fit indices, path coefficients, critical ratios (C.R.), and R^2^ values between the hypothesized and alternative structures. The results, detailed in the "Path Analysis" section, are as follows:

**Path Reversal of H3:** When the path was reversed to "Perceived Service Quality → Perceived Social Value," the global fit indices remained stable, but the "Crowding Stress →Perceived Service Quality" path lost its significance. This indicates a disruption in the model’s internal structural integrity and a decline in parameter identification power.

**Path Reversal of H5:** Reversing the direction to "Perceived Social Value →Perceived Crowding" yielded no significant improvement in the overall goodness-of-fit.

**Path Reversal of H6:** Reversing the direction to "Loyalty →Perceived Crowding" resulted in a statistically non-significant path, suggesting that the causal logic in this direction is not empirically supported.

In summary, the competing model analysis demonstrates that none of the alternative structures offer superior statistical robustness or reliability in parameter identification. Given the theoretical consistency and empirical fit, the **initial hypothesized model** remains the most parsimonious and valid framework.

### Reviewers' comments:

5. Conceptual and empirical contradictions

Tension between high crowding perceptions and positive experience.

Descriptively, the item “The congestion in Tianshui has impacted my travel experience” has M = 3.37 (above midpoint) with high SD = 1.17, indicating many tourists felt impacted by congestion. Yet the discussion strongly emphasises that crowding “did not erode loyalty” and that congestion was “acceptable”. This interpretation is plausible but somewhat optimistic and not backed by segment-level analysis (e.g., high‑vs‑low crowding perception groups).

### Response

We appreciate the reviewer’s acute observation. Our further analysis revealed a **U-shaped non-linear correlation** between perceived crowding and tourist loyalty. A scatter plot and linear fit between loyalty and crowding stress factor scores yielded an R^2^ of only **0.037**, indicating that a linear model has extremely weak explanatory power.

Examining the sample distribution, we observed that in the **low-stress interval**, tourist loyalty remains at a high and stable level with low variance. As crowding stress shifts to the **medium interval**, the average loyalty level drops significantly. Paradoxically, when crowding stress reaches the **high interval**, the mean loyalty level consistently rebounds. This suggests a distinct U-shaped trajectory where loyalty first declines and then ascends as pressure increases.

Furthermore, the **dispersion (variance)** of loyalty in the medium-stress interval is significantly higher than at either extreme, forming an inverted triangular scatter pattern. This characteristic of **fluctuation heterogeneity** reinforces our judgment that the relationship is not a simple linear one, but rather a potential **quadratic (non-linear) relationship**.

Fig 2. Scatter plot of factor scores for perceived crowding and loyalty

To further examine the potential non-linear relationship between perceived crowding and loyalty, this study constructed a baseline linear regression model (Model 1) and a quadratic non-linear regression model (Model 2). The estimation results are presented in Table 6.

Table 2. Regression results for the relationship between perceived crowding and loyalty

| Variables | Model 1 | | Model 2 | |
| --- | --- | --- | --- | --- |
|  | β | SD | β | SD |
| Constant | 3.497*** | 0.126 | 4.878*** | 0.292 |
| PC | 0.170*** | 0.045 | -1.036*** | 0.236 |
| PC^2^ |  |  | 0.239*** | 0.046 |
| R^2^ | 0.037 | | 0.102 | |
| F | 14.328*** | | 21.205*** | |

The analysis results indicate that compared to the baseline linear model, the non-linear model—which incorporates the quadratic term of perceived crowding—demonstrated a significant improvement in goodness-of-fit, with R^2^ increasing from 0.037 to 0.102, substantially enhancing the model's explanatory power.

According to the regression coefficients of the quadratic model, the linear term of perceived crowding was significantly negative (β_1_ = -1.036, p < 0.001), while the quadratic term was significantly positive (β_2_ = 0.239, p < 0.001). The calculated turning point (X*) was 2.17, which falls within the measured range of perceived crowding (95% confidence interval: [1.85, 2.49]). Furthermore, the slopes at both ends of the interval were significantly negative and positive, respectively, satisfying the requirements for a U-shaped relationship. These findings confirm a significant U-shaped non-linear relationship between perceived crowding and customer loyalty.

the potential explanatory mechanism is that differences in tourists' psychological carrying capacities serve as the core driver of this U-shaped relationship. Specifically, tourists with high psychological carrying capacity view crowding as an inherent characteristic of "Internet-famous" destinations; they recognize and accept the atmosphere created by the crowds, resulting in minimal interference and a smooth experience, thus maintaining loyalty at a high level. For tourists with low psychological carrying capacity who are sensitive to crowding, although they visit driven by the bandwagon effect and "fear of missing out" (FOMO), the crowding significantly interferes with their visit, leading to psychological stress and avoidance tendencies. However, such tourists may still have high recognition of the destination's core attractions. Under these dual forces, they may develop a compensatory revisit intention—a desire to return at a different time to complete an "incomplete experience"—which likewise keeps their loyalty at a high level. For tourists with medium psychological carrying capacity, the perceived pressure of crowding is also at a moderate level. At this point, the crowding may disrupt their pace and experience quality, exceeding their comfort threshold without reaching the extreme pressure required to trigger a compensatory motive, thus leading them into a "moderate experience trap." These tourists neither gain sufficient satisfaction nor develop a strong compensatory revisit intention, resulting in relatively lower loyalty.

### Reviewers' comments:

Claim that internet-famous status influences loyalty “only through indirect pathways” is overstated.

The study does not directly measure “internet-famous status” as a construct; rather, it uses perceived social value and perceived crowding as proxies, in a single destination already selected for being viral. To claim that “internet-famous destination status influences loyalty only through indirect pathways” extrapolates beyond what the data actually measure.

### Response

We have replaced "Internet Fame Status" with the theoretically grounded term **"Perceived Social Value."** Additionally, we have refined our model to specify that in the context of viral tourism, **Perceived Social Value is linked to loyalty exclusively through the full mediation of Perceived Service Quality.** This underscores that social prestige must be anchored by tangible service excellence to generate long-term tourist loyalty.

### Reviewers' comments:

Quasi-experimental language without design.

The paper describes Tianshui as a “quasi-experimental” setting and references “viral cycles,” but there is no temporal or comparative design to justify this term. This can be perceived as overselling the methodological strength.

### Response

We have removed the reference to a **"quasi-experimental"** setting and instead emphasized a **"case study"** approach.

### Reviewers' comments:

Other comments:

Figures not fully described: The text refers to Fig 1 (hypotheses model) and Fig 2 (estimated results) and lists them again as supporting information S1 and S2, but in the manuscript format shown, the figures are not visible and their content is only briefly mentioned. For review, paths, coefficients, and error terms in the figures should be clearly and consistently reported.
Ethical procedures described only briefly and without institutional approval information.
The paper notes verbal consent, anonymity, and parental consent for minors, but there is no mention of ethics committee approval or waiver, which many journals now require explicitly

### Response

**Inserted the figure into the paper.**

**Ethical Statement:** This study has been formally granted an **ethics review waiver** by the **Academic Ethics Committee of Lanzhou University of Finance and Economics**.

## Reviewer #3:

### Reviewers' comments:

1.Title: you'd have to add method used into your title, like""

### Response

The methodology used has been incorporated, and the title has been revised to: **"Research on Tourist Loyalty of Internet-famous Destinations Based on Structural Equation Modeling: Perceived Service Quality, Perceived Social Value, and Crowding Effects."**

### Reviewers' comments:

2.Abstract: I suggest that all the highlights should be bolded and with the first letter capitalized. This will make it easier for readers to understand at a glance.

### Response

Important terms have been **bolded** in accordance with the suggestions.

### Reviewers' comments:

3.Keywords should be added into title page

### Response

The revisions have been made in accordance with the comments.

### Reviewers' comments:

4.The content of lines 178 to 194 is redundant. Delete this part or reduce it as much as possible to the description style of a scientific article. Now it seems more like a piece of news.

### Response

This section has been rewritten.

### Reviewers' comments:

5.The entire "results" section should be a coherent whole, or it can be categorized using sub-sections or levels 2 and 3 headings. Descriptive statistics should include Table 1.

### Response

The revisions have been made in accordance with the comments.

### Reviewers' comments:

6.The first step in conducting a structural equation model should be the examination of common method variance to ensure the validity of the questionnaire. Please include this.

### Response

To ensure the rigor of our empirical results, we conducted a Common Method Bias test. The procedures and results have been detailed in the "Measurement Model Testing" section:

A Harman’s single-factor test was conducted as a preliminary assessment. The unrotated exploratory factor analysis (EFA) of all scale items revealed that the first principal factor explained 41.593% of the variance. As this slightly exceeded the 40% threshold, it suggested the potential presence of common method variance (CMV). Following the recommendations of Tang et al. (2020), the CFA marker variable technique was employed to further verify the severity of this bias. A marker variable (M), unrelated to the research variables, was added to the CFA model to construct four sequential models: the baseline model (assuming no CMV), Model C (assuming CMV with equal influence on all indicators), Model U (assuming CMV with varying influence on all indicators), and Model R (assuming CMV without affecting correlations between variables).

The chi-square differences between these models were estimated and compared. A significant chi-square difference was observed between the baseline model and Model C ( Δχ² (Δdf = 1) = 7.762,p = 0.005), indicating the presence of CMV. Furthermore, the difference between Model C and Model U was significant (Δχ²(Δdf = 11) =19.703, p = 0.049), showing that CMV influenced the indicators differently. However, no significant difference was found between Model U and Model R (Δχ² ( Δdf = 6 )=0.968, p =0.987). In summary, while slight common method variance exists in the data, it does not constitute a severe common method bias (CMB) and will not substantially interfere with the research results or hypothesis testing. Thus, the data are suitable for subsequent structural model analysis.

Tang D D, Wen Z L. Statistical Approaches for Testing Common Method Bias: Problems and Suggestions. Journal of Psychological Science, 2020, 43(1): 215-223.

### Reviewers' comments:

7.The "discussion" section should thoroughly analyze all the results. The length needs to be significantly increased. Currently, the entire article appears more like a draft rather than a complete paper.Maybe this paper will help you (10.1038/s41598-025-15612-6).

### Response

We appreciate the references you provided, which have been very inspiring. We have rewritten the "Discussion" section to include a more in-depth analysis of the results.

## Reviewer #4:

### Reviewers' comments:

First, the theoretical contribution appears incremental, as relationships such as experience quality influencing loyalty are already well established; therefore, the authors should clearly articulate what is conceptually new—particularly whether the novelty lies in the “internet-famous destination” construct, the moderating role of comment interaction, or the interpretation of crowding effects.

### Response

The "Discussion" section elucidates the theoretical significance of this study as follows:

**Robustness of the Service-Loyalty Link:** It validates the robustness of the relationship between perceived service quality and tourist loyalty within the emerging context of "Internet-famous" destination tourism.

**Indirect Role of Social Value:** It confirms the indirect impact of perceived social value on loyalty, mediated through quality experience.

**Non-linear Crowding Effects:** It uncovers a non-linear relationship between perceived crowding and loyalty in social-media-driven tourism settings. Specifically, as perceived crowding increases, loyalty exhibits a "U-shaped" trend—initially declining before rebounding—rather than the continuous decline suggested by traditional paradigms.

**Hedonic Decay in Viral Marketing:** It identifies that excessive online marketing of "viral cities" may trigger **"hedonic decay"** among tourists. This phenomenon weakens the transformation of perceived service value into long-term loyalty, potentially jeopardizing the destination's sustainable tourist traffic.

### Reviewers' comments:

Relatedly, the term “internet-famous destination” requires a stronger academic definition with measurable attributes and clearer differentiation from popular or overtourism destinations.

### Response

Following the definition of "Internet-famous" cities by **Zou S. ( 2025)**, this study defines an "Internet-famous" destination as a city that has gained immense popularity through social media platforms (such as Douyin and Xiaohongshu) and has evolved into a **viral tourism destination**.

Zou S. Transcoding a wanghong city: Mediatized culturalization of urban places in China. Media, culture & society, 2025, 47(1): 22-40.

### Reviewers' comments:

A major methodological concern is the use of a single-item measure for perceived comment interaction (“I think the comment section of the Tianshui Malatang short video is very interesting”), which raises questions about construct validity and should either be justified with literature or acknowledged as a limitation.

### Response

We appreciate your professional suggestion. We agree that multi-item scales offer significant advantages in capturing complex constructs. According to Moon & Kim (2001), perceived playfulness is defined as an intrinsic motivation reflecting a "flow experience" during user-system interaction [79], which constitutes an intuitive evaluation. Video comment sections frequently serve as hubs for secondary creation and primary sources of Internet memes; the abundant humor and social resonance found therein can generate significant emotional pleasure and sensory satisfaction [80], aligning with the hedonic nature of this construct. In social media-driven tourism contexts, following viral videos is a fundamental behavior of tourists, providing them with a clear cognitive basis to assess the interest level of comment sections. Following Bergkvist et al. (2007), for well-defined variables with concrete attributes, a single-item measure can effectively capture respondents' authentic perceptions. Therefore, this study employed a single-item indicator for the moderating variable to meet the statistical requirements for moderation analysis, treating it as a fixed error term to examine its moderating effect. As a sophisticated affective construct, **perceived fun (playfulness)** potentially encompasses multiple dimensions, such as humor, novelty, and social interaction. While a single-item measure can reflect overall perception, it may lack the granularity required to characterize deep-seated psychological structures. Consequently, future research could focus on developing a multidimensional scale for fun to further validate the robustness of the conclusions presented in this study.

Moon J W, Kim Y G. Extending the TAM for a World-Wide-Web context. Information & management, 2001, 38(4): 217-230.

Bergkvist L, Rossiter J R. The predictive validity of multiple-item versus single-item measures of the same constructs. Journal of marketing research, 2007, 44(2): 175-184.

### Reviewers' comments:

The sample is heavily skewed toward young respondents aged 18–25 with relatively low income, potentially limiting generalizability; the authors should discuss generational bias and consider positioning the findings within Gen Z travel behavior.

### Response

We fully concur with your insightful comments and have addressed this issue in the **"Research Limitations"** section of the manuscript as follows: **Methodological limitations in sampling.** This study utilized convenience sampling for data collection, which presents inherent limitations regarding the representativeness of the tourist population. Specifically, the sample exhibits a tendency toward homogeneity, predominantly consisting of "highly educated young people." While this demographic profile aligns with previous research on similar viral cities like Zibo , and—as noted by Jager et al. (2017)—homogeneous samples can enhance internal validity by minimizing interference from sub-group extraneous variables [94], it nevertheless restricts the generalizability of the findings to the broader tourism market. Furthermore, data were collected at fixed locations within major scenic spots in Tianshui, which may introduce spatial bias. Finally, due to constraints in manuscript length and sample size, this study did not perform multi-group analysis, potentially overlooking significant variations in research outcomes across different sub-groups.

Jager, J., Putnick, D. L., & Bornstein, M. H. II. More than just convenient: The scientific merits of homogeneous convenience samples. Monographs of the society for research in child development, 2017, 82(2), 13-30.

### Reviewers' comments:

Additionally, because data were collected within a short holiday window using on-site questionnaires, the study captures loyalty intention rather than actual loyalty, and this distinction should be explicitly acknowledged along with the constraints of a cross-sectional design.

### Response

**Construct Definition:** We have clarified in the "Variable Measurement" section that the loyalty measured in this study refers specifically to **loyalty intention**.

**Common Method Bias (CMB):** Acknowledging the limitations inherent in cross-sectional designs, we conducted a **Common Method Bias test**. The results indicate that while some common method variance exists, it does not exert a substantial interference on the research findings or the hypothesis testing. Therefore, the data are suitable for subsequent structural model analysis.

**Research Limitations:** We have added a targeted discussion regarding the constraints of the **cross-sectional design** in the "Research Limitations" section.

### Reviewers' comments:

The deletion of item X9 due to weak loading (0.445) is statistically acceptable but warrants further explanation regarding scale stability.

### Response

After the removal of certain items, the model passed the **Confirmatory Factor Analysis (CFA)**, demonstrating satisfactory **convergent and discriminant validity**. This indicates that the refined model possesses sound **construct validity** and is statistically acceptable.

### Reviewers' comments:

The finding that crowding does not reduce loyalty is particularly interesting but under-theorized; expanding the discussion through concepts such as social proof, bandwagon effects, or FOMO could strengthen the contribution.

### Response

Based on **Psychological Carrying Capacity Theory**, the non-linear U-shaped relationship between perceived crowding stress and tourist loyalty at "Internet-famous" destinations can be explained as follows:

**High Psychological Carrying Capacity Segment:** Crowding is an inherent byproduct of viral popularity. Tourists with a high psychological carrying capacity perceive crowding as a defining characteristic of the "Internet-famous" atmosphere. They accept and even embrace this density, experiencing minimal disruption and high-quality flow. Consequently, their travel experience remains superior, maintaining **loyalty at a high level**.

**Low Psychological Carrying Capacity Segment (The "Compensation" Effect):** Crowding-sensitive tourists are often driven to visit by the **Bandwagon Effect** and **Fear of Missing Out (FOMO)**. While high crowding significantly disrupts their onsite experience and generates substantial stress, these tourists still affirm the destination's core appeal. Under the influence of **Cognitive Dissonance**, they rationalize their travel decision by inflating their psychological evaluation of the destination's value. This dual mechanism fosters a "revisit to compensate" motive—where tourists feel the current visit was incomplete and plan to return during off-peak periods—thereby paradoxically **sustaining high loyalty**.

**Moderate Psychological Carrying Capacity Segment (The "Medium Experience Trap"):** For tourists with moderate carrying capacity, perceived stress is also at a medium level. Here, crowding has sufficiently disrupted the rhythm and quality of the tour, exceeding their comfort threshold but without reaching the extreme levels that trigger compensatory rationalization. These tourists fall into a **"medium experience trap"**: they neither derive full satisfaction nor develop strong compensatory revisit intentions, resulting in the **lowest levels of loyalty**.

### Reviewers' comments:

From a presentation perspective, the manuscript would benefit from language polishing, reduced repetition (especially of “internet-famous destination”), stronger synthesis in the literature review rather than descriptive listing, and greater methodological transparency regarding random street intercept procedures and enumerator training.

### Response

The relevant revisions have been made and the aforementioned content has been added; details regarding investigator training have been incorporated into the "Data Collection" section.

### Reviewers' comments:

Practical implications should be expanded to provide more actionable recommendations for destination marketers, and the limitations section should be broadened to include the single-destination context, single-item moderator, cross-sectional data, and age-skewed sample.

### Response

Statements regarding the limitations of the aforementioned issues have been added to the "Research Limitations" section.

## Reviewer #5:

### Reviewers' comments:

the scientific novelty of the work should be presented in the abstract;

### Response

The abstract has been rewritten and relevant content has been added.

### Reviewers' comments:

the discussion section is poorly written; it should present the validation/invalidation of the established hypotheses and link them to the conclusions of relevant references;

### Response

The "Discussion" section has been rewritten and relevant content has been added.

### Reviewers' comments:

authors should clearly present the contribution of their research to the academic literature in the field and to the economic environment (stakeholders in the tourism sector).

### Response

Relevant content has been added to the "Discussion" section.
