# Supplementary material for: Structural equation modeling of tourist loyalty in Tianshui, China: Perceived service quality, perceived social value, and crowding effects
Source: PLoS One. 2026 Oct 7;21(10):e0357597. doi: 10.1371/journal.pone.0357597 (PMC13645047; doi:10.1371/journal.pone.0357597)
Supplement: Supplementary file 5 [file pone.0357597.s006.docx]

# **Response to Reviewers**

## ****1.**** ****Reviewer #1****

### Reviewers' comments:

Study on Tourist Loyalty at Internet-Famous Destinations Based on Structural Equation Modeling: Perceived Service Quality, Perceived Social Value, and Crowding Effects

Suggested title: Structural equation modeling of tourist loyalty in Tianshui, China: Perceived service quality, perceived social value, and crowding effects

### Response：

The title of the manuscript has been revised to:Structural equation modeling of tourist loyalty in Tianshui, China: Perceived service quality, perceived social value, and crowding effects

### Reviewers' comments:

ABSTRACT

• In line 24, delete (SEM)

• Use of “we” in line 24 should be delete

• There is no well defined method explained

• There is lack of stated findings

• This is not a discussion session to be drawing inferences

### Response：

The abbreviation (SEM) has been removed, and the pronoun "we" has been eliminated throughout. We have comprehensively revised the abstract, provided clear descriptions of the research methodology, listed all empirical findings of this study, and removed analytical inferences within the abstract. Please refer to the revised manuscript for details.

### Reviewers' comments:

Introduction

• Provide reference to support the statement from line 39 to line 42

• From line 100 to line 106, no reference

### Response：

We have added the following references to support the arguments in Lines 39–42 of the manuscript：

Li K, Chen Z. From abroad to home: unveiling the surge in chinese domestic travel through the lens of affective economies. Journal of Tourism and Cultural Change, 2025, 23(4): 345-366.

In Lines 100–106 of the original manuscript, we introduce the Expectation-Confirmation Theory to reinterpret the effects of online information and offline experience on tourists’ perceptions, and two additional references are supplemented for theoretical support：

Oliver R L. A cognitive model of the antecedents and consequences of satisfaction decisions. Journal of marketing research, 1980, 17(4): 460-469.

Narangajavana Kaosiri Y, Callarisa Fiol L J, Moliner Tena M Á, et al. User-generated content sources in social media: A new approach to explore tourist satisfaction. Journal of Travel Research, 2019, 58(2): 253-265.

### Reviewers' comments:

Research Hypotheses

• I sincerely do not see the need to have a preamble which includes line 162 to line 174

• Again, from line 1774 to line 184

• Also, from line 187 to line 193

• From line 196 to line 209 is redundant

• From line 214 to line 221 is also not necessary

• From line 224 to 244 delete

• Having up to seven (7) hypothesis in one study is not an ideal thing to do. Kindly reduce the hypothesis to just two or at most three

• Again, the stated hypothesis, there are null or alternatives

### Response：

We sincerely appreciate the constructive comments from the reviewer. The reviewer suggested deleting all textual discussions in the "Research Hypotheses" section, retaining only the hypotheses, and consolidating the seven hypotheses into two to three. Following careful discussion and thorough deliberation regarding this suggestion, we respond as follows:

After thoroughly reviewing the narrative in the "Research Hypotheses" section, we found instances of textual redundancy and content overlap with the "Research Framework," which indeed required streamlining—likely the intended purpose of the reviewer's suggestion. However, the primary function of this section is to provide the theoretical basis for the causal paths between variables and to propose the research hypotheses for each path in the structural equation model. Deleting all introductory text would leave the hypothesized relationships lacking logical foundation, creating obstacles for readers to comprehend the paper. Therefore, we have substantially streamlined the narrative in the "Research Hypotheses" section by removing redundant wording and retaining only concise text to describe the theoretical derivation of the relationships, thereby ensuring structural integrity and readability.

The seven research hypotheses in this study correspond to seven distinct paths among variables in the structural equation model. Reducing the number of hypotheses to two or three would enhance conciseness and offer a quick overview of the study. However, doing so would cause each hypothesis to cover multiple paths belonging to different causal chains, potentially rendering the hypotheses ambiguous and hindering clear expression. To address the issue of having overly tedious hypotheses without losing clarity, we reorganized the original hypotheses into three logical categories and grouped them accordingly in the manuscript: (1) relationships among perceived service quality, perceived social value, and loyalty (H1–H3); (2) relationships between perceived crowding and the former three variables (H4–H6); and (3) the moderating effect of perceived playfulness (H7). This approach maintains the clarity of all seven paths while improving logical structure and reducing the reader's burden. Please refer to the revised manuscript for details.

Finally, due to space limitations, H1–H7 listed in the text are alternative hypotheses; the corresponding null hypotheses are not repeatedly listed as they are implicitly assumed following standard hypothesis testing paradigms. Additionally, in the “Hypothesis testing–Path Analysis” section, we have removed the “Results” column from Table 4 and used standard statistical phrasing—such as “reject the null hypothesis” and “fail to reject the null hypothesis”—when reporting hypothesis testing conclusions in the main text.

### Reviewers' comments:

Materials and methods

Data collection

• In line 274, the use of interviews is not appropriate with the study

• In line 279, the use of 375 valid questionnaires were recovered meant what?

### Response：

The above comments were originally in the same paragraph; an overall adjustment has been made to this paragraph, correcting vague meanings and inappropriate phrasing. Please refer to the revised manuscript for details.

### Reviewers' comments:

• Provide a reference to support the range of value for the use of the Cronbach’s alpha coefficients. You may refer to Taber’s interpretations

### Response：

Added Taber's paper as supporting literature:

Taber K S. The use of Cronbach’s alpha when developing and reporting research instruments in science education. Research in science education, 2018, 48(6): 1273-1296.

### Reviewers' comments:

Results

• In line 360, the use of Descriptive statistical analysis should be deleted

### Response：

"Descriptive statistical analysis" has been removed.

### Reviewers' comments:

• In line 441, Table 2 should be moved to materials and methods if indeed it should be shown

### Response：

Regarding the section placement of Table 2, we have thoroughly reviewed and optimized it. Our response to this comment is as follows:

The metrics reported in the original Table 2 can be divided into three categories: descriptive statistics (the mean and SD for each item, representing the analysis of sample data); internal consistency test results (Cronbach’s α for each dimension, testing whether items under the same dimension are consistent in the basic data); and CFA results (standardized factor loadings for each item, as well as CR and AVE for each dimension, used to evaluate single-item convergence, overall dimensional consistency, and overall dimensional convergence). The original manuscript combined multiple types of metrics into a single table with the initial intention of saving space and avoiding duplicate tables, but this resulted in an overly cluttered table. However, moving Table 2 entirely to the "Materials and Methods" section would introduce a substantial amount of data analysis content into that section, blurring the distinct functions of each section, while also causing the CFA results to appear prematurely, thereby diminishing reading coherence.

Considering the above factors, we retained the location of Table 2 while streamlining its content by removing the Mean and SD columns, presenting text descriptions of the mean and SD for key metrics during the descriptive statistical analysis instead. This addresses the issue of Table 2 being overly cluttered without affecting the reporting of core data, while maintaining the coherence and logical rigor of the empirical testing in the "Results" section.

### Reviewers' comments:

In Table 3, provide a legend for the acronyms used

### Response：

All abbreviations in Table 3 have been changed to their full terms.

### Reviewers' comments:

Discussion

• In line 551, the discussion of findings should be deleted

### Response：

Content related to the study results has been deleted from the "Discussion" section.

### Reviewers' comments:

Conclusion and Future Research

• In line 666, the heading should be deleted

### Response：

the heading is already been deleted.

### Reviewers' comments:

General Comments

• There is significant improvement in the manuscript

• Authors may have a look at my humble suggestions to improve the content of the manuscript

### Response：

Thank you very much for your thorough and helpful comments. We have carefully considered all of your suggestions and revised the manuscript accordingly.

## ****2. Reviewer #4****

### Reviewers' comments:

The manuscript has undergone significant revision and demonstrates noticeable improvement. The responses to reviewers are comprehensive, and most of the previous concerns have been adequately addressed. The revised paper presents a clearer research framework, improved methodology, and stronger discussion. Nevertheless, several issues remain that should be addressed to further strengthen the manuscript.

### Response：

We greatly appreciate your positive evaluation of the revised manuscript. We have conducted a comprehensive self-examination and further polished the manuscript throughout the text to improve its quality.

As you mentioned that several issues remain to be addressed, we would highly appreciate it if you could provide specific comments on these remaining concerns. We are ready to make further targeted revisions strictly following your suggestions.

## ****3. Reviewer #5****

The authors have made substantial improvements to the manuscript, and I consider it suitable for publication now. Good luck!

### Response：

Thank you very much for your positive assessment and recognition of our revisions. We highly value your time and efforts spent on this manuscript.
