# Supplementary material for: Structural equation modeling of tourist loyalty in Tianshui, China: Perceived service quality, perceived social value, and crowding effects
Source: PLoS One. 2026 Oct 7;21(10):e0357597. doi: 10.1371/journal.pone.0357597 (PMC13645047; doi:10.1371/journal.pone.0357597)
Supplement: Peer review history

# Peer review: Decision Letter 0

**DOI:** 10.1371/journal.pone.0357597.r001

**Authors:** Bo Pu

15 Feb 2026

PONE-D-26-02054

Can Becoming an Internet-Famous Destination Influence Tourist Loyalty?

Dear Dr. Huang,

Thank you for submitting your manuscript to PLOS ONE. After careful consideration, we feel that it has merit but does not fully meet PLOS ONE’s publication criteria as it currently stands. Therefore, we invite you to submit a revised version of the manuscript that addresses the points raised during the review process.

Please submit your revised manuscript by Apr 01 2026 11:59PM. If you will need more time than this to complete your revisions, please reply to this message or contact the journal office at plosone@plos.org. When you're ready to submit your revision, log on to https://www.editorialmanager.com/pone/ and select the 'Submissions Needing Revision' folder to locate your manuscript file.

- A letter that responds to each point raised by the academic editor and reviewer(s). You should upload this letter as a separate file labeled 'Response to Reviewers'.
- A marked-up copy of your manuscript that highlights changes made to the original version. You should upload this as a separate file labeled 'Revised Manuscript with Track Changes'.
- An unmarked version of your revised paper without tracked changes. You should upload this as a separate file labeled 'Manuscript'.

If you would like to make changes to your financial disclosure, please include your updated statement in your cover letter. Guidelines for resubmitting your figure files are available below the reviewer comments at the end of this letter.

If applicable, we recommend that you deposit your laboratory protocols in protocols.io to enhance the reproducibility of your results. Protocols.io assigns your protocol its own identifier (DOI) so that it can be cited independently in the future. For instructions see: https://journals.plos.org/plosone/s/submission-guidelines#loc-laboratory-protocols. Additionally, PLOS ONE offers an option for publishing peer-reviewed Lab Protocol articles, which describe protocols hosted on protocols.io. Read more information on sharing protocols at https://plos.org/protocols?utm_medium=editorial-email&utm_source=authorletters&utm_campaign=protocols.

We look forward to receiving your revised manuscript.

Kind regards,

Bo Pu, Ph.D.

Academic Editor

PLOS One

Journal Requirements:

When submitting your revision, we need you to address these additional requirements.

1. Please ensure that your manuscript meets PLOS ONE's style requirements, including those for file naming. The PLOS ONE style templates can be found at

https://journals.plos.org/plosone/s/file?id=wjVg/PLOSOne_formatting_sample_main_body.pdf and

https://journals.plos.org/plosone/s/file?id=ba62/PLOSOne_formatting_sample_title_authors_affiliations.pdf

2. In the ethics statement in the Methods, you have specified that verbal consent was obtained. Please provide additional details regarding how this consent was documented and witnessed, and state whether this was approved by the IRB

3. We note that the grant information you provided in the ‘Funding Information’ and ‘Financial Disclosure’ sections do not match.

When you resubmit, please ensure that you provide the correct grant numbers for the awards you received for your study in the ‘Funding Information’ section.

4. Thank you for stating the following financial disclosure:

“Natural Science Foundation Project of Gansu Province, China, under Grant grant number 24JRRA1005; the Research Foundation Project of Lanzhou University of Finance and Economics, China, under Grant Lzufe2024C-016”

Please state what role the funders took in the study.  If the funders had no role, please state: "The funders had no role in study design, data collection and analysis, decision to publish, or preparation of the manuscript."

If this statement is not correct you must amend it as needed.

Please include this amended Role of Funder statement in your cover letter; we will change the online submission form on your behalf.

5. Thank you for stating the following financial disclosure:

“Natural Science Foundation Project of Gansu Province, China, under Grant grant number 24JRRA1005; the Research Foundation Project of Lanzhou University of Finance and Economics, China, under Grant Lzufe2024C-016”

We note that one or more of the authors is affiliated with the funding organization, indicating the funder may have had some role in the design, data collection, analysis or preparation of your manuscript for publication; in other words, the funder played an indirect role through the participation of the co-authors. If the funding organization did not play a role in the study design, data collection and analysis, decision to publish, or preparation of the manuscript and only provided financial support in the form of authors' salaries and/or research materials, please do the following:

1. Review your statements relating to the author contributions, and ensure you have specifically and accurately indicated the role(s) that these authors had in your study. These amendments should be made in the online form.

2. Confirm in your cover letter that you agree with the following statement, and we will change the online submission form on your behalf:

“The funder provided support in the form of salaries for authors [insert relevant initials], but did not have any additional role in the study design, data collection and analysis, decision to publish, or preparation of the manuscript. The specific roles of these authors are articulated in the ‘author contributions’ section.

6. PLOS requires an ORCID iD for the corresponding author in Editorial Manager on papers submitted after December 6th, 2016. Please ensure that you have an ORCID iD and that it is validated in Editorial Manager. To do this, go to ‘Update my Information’ (in the upper left-hand corner of the main menu), and click on the Fetch/Validate link next to the ORCID field. This will take you to the ORCID site and allow you to create a new iD or authenticate a pre-existing iD in Editorial Manager.

7. Please include your full ethics statement in the ‘Methods’ section of your manuscript file. In your statement, please include the full name of the IRB or ethics committee who approved or waived your study, as well as whether or not you obtained informed written or verbal consent. If consent was waived for your study, please include this information in your statement as well.

8. We notice that your supplementary figures are included in the manuscript file. Please remove them and upload them with the file type 'Supporting Information'. Please ensure that each Supporting Information file has a legend listed in the manuscript after the references list.

9. If the reviewer comments include a recommendation to cite specific previously published works, please review and evaluate these publications to determine whether they are relevant and should be cited. There is no requirement to cite these works unless the editor has indicated otherwise.

Additional Editor Comments:

please revise this manuscript according to the reviewers' comments.

[Note: HTML markup is below. Please do not edit.]

Reviewers' comments:

Reviewer's Responses to Questions

**Comments to the Author**

1. Is the manuscript technically sound, and do the data support the conclusions?

Reviewer #1: Partly

Reviewer #2: Partly

Reviewer #3: Partly

Reviewer #4: Partly

Reviewer #5: Yes

**********

2. Has the statistical analysis been performed appropriately and rigorously?

Reviewer #1: No

Reviewer #2: Yes

Reviewer #3: Yes

Reviewer #4: Yes

Reviewer #5: Yes

**********

3. Have the authors made all data underlying the findings in their manuscript fully available?

The PLOS Data policy

Reviewer #1: No

Reviewer #2: No

Reviewer #3: Yes

Reviewer #4: Yes

Reviewer #5: Yes

**********

4. Is the manuscript presented in an intelligible fashion and written in standard English?

Reviewer #1: Yes

Reviewer #2: Yes

Reviewer #3: Yes

Reviewer #4: Yes

Reviewer #5: Yes

**********

Reviewer #1: Kindly refer to the comments and address them to enhance the quality of the work. Once this is done and the work is improved significantly, then it will be a good paper. As it stands now, the paper is not good to be published

Reviewer #2: Dear authors,

I hope the following comments will be helpful:

1. Theoretical framing and hypotheses

Conceptualisation of “perceived social value” is narrow and overlaps with other constructs.

The scale items capture social attention, popularity, making friends, and self-confidence (X1–X4), which mix social status, social relationship gains, and self-related benefits under one factor, yet the theory section does not justify why these heterogeneous aspects belong to a single latent construct or how it differs from social image/value, self-enhancement, or hedonic value.

Operationalisation does not fully reflect SOR and UGT arguments.

The SOR framing suggests multiple types of stimuli (UGC, authenticity of viral atmosphere, congruence between online and offline experience), but the model only includes perceived crowding and perceived social value as “stimuli”, with experience quality as “organism” and loyalty as “response”; key digital elements (e.g., online authenticity, congruence, expectations) are discussed conceptually but not measured.

H1 is not logically integrated with later mediation.

The hypothesis states that perceived social value directly increases loyalty (H1), then the mediation section argues that experience quality fully mediates this relationship and that social value has no direct effect on loyalty. This is not just a statistical outcome; it contradicts the initial claim that social value is a direct antecedent of loyalty, and there is no ex-ante rationale for why the effect should become fully indirect.

Contradiction in the treatment of H6.

H6 hypothesises “no significant influence” of perceived crowding on loyalty. The structural model finds a non-significant path (β = 0.044, p > 0.05) and labels H6 as “Accept.”

Accepting a null hypothesis is statistically contentious; usually one “fails to reject” it and would need power analysis or equivalence testing to support a claim of “no effect”. This should be reformulated or justified more rigorously.

Moderation by “perceived comment interaction” is under-theorised and minimally measured.

The moderation hypothesis (H7) is based on Uses-and-Gratifications Theory, but the moderator is measured by a single item (“I think the comment section … is very interesting”), which mainly reflects entertainment rather than interaction or participation (no frequency, depth, or type of engagement).

This weak alignment between theory (active co‑creation, gratifications) and measurement (interest in comments) raises construct validity concerns.

2. Research design and external validity

Single destination and very narrow time window.

Data are collected only in Tianshui during one holiday (Qing-Ming, 4–6 April 2024), within a specific boom period. This severely limits generalisability to other “internet-famous” destinations, off‑peak periods, or other types of viral events. The paper calls the setting a “quasi-experimental window” but does not employ any quasi-experimental design (no pre‑post, control group, or natural experiment structure).

Sample composition may bias results but is not critically discussed.

The majority are 18–25 years old (58.7%), low-income, and highly educated (72% bachelor/college diploma), which is not representative of the broader tourist market. Given that social media use and comment interaction are age- and education-sensitive, the findings may primarily describe young social-media-savvy tourists; this limitation is not sufficiently emphasised.

Potential common method bias is not addressed.

All constructs (perceived social value, experience quality, crowding, loyalty, and the moderator) are measured via self-report, in the same questionnaire, at a single time point; yet there is no mention of Harman’s one-factor test, CFA marker-variable tests, or other checks for common method variance.

Cross-sectional data used to infer directional/causal relations.

The study interprets paths as “influences” (e.g., perceived social value → experience quality; experience quality → loyalty), but the design is purely cross-sectional and observational; no temporal separation or experimental manipulation supports causal claims. A Q1 journal would expect a more cautious interpretation.

3. Measurement and model specification

Weakness in perceived crowding measurement.

Perceived crowding is measured with only two items: “congestion impacted my travel experience” and “I should avoid hotspots and come back later” (X10–X11).

These conflate: perceived density (crowding), affective reaction, and behavioural intention (avoidance).

Thus, the construct is not a pure measure of “crowding” and overlaps conceptually with experience quality and loyalty/behavioural intentions, which may distort path estimates.

Marginal reliability for perceived crowding.

Cronbach’s α and composite reliability for perceived crowding are both 0.699, just below or at the threshold; the text states they “meet or approach” 0.70, but this borderline reliability, combined with only two items, should be more critically discussed.

Ad-hoc deletion of X9 may affect construct validity.

Item X9 (“Long queuing time for Malatang negatively affected my tourism experience”) is deleted because of a low factor loading (0.445), improving fit indices. However:

Theoretical implications are not fully examined (queuing is central to viral food destinations; removing it may truncate the experience-quality dimension).

No cross-validation is performed; the deletion is based on a single split-sample CFA.

The crowding construct then no longer covers queueing, which is a key aspect of “viral” crowding.

Use of split-sample EFA/CFA with moderate N.

With N = 375, the authors split into N = 167 and N = 208 for EFA and CFA. While methodologically neat, this reduces power and stability in both analyses; a Q1 journal might ask for additional validation (e.g., full-sample CFA, or cross-validation with an independent sample).

Single-item moderator and lack of measurement model integration.

Perceived comment interaction is treated outside the SEM and estimated via hierarchical regression using factor scores, rather than as a latent construct within the SEM framework. This introduces:

� inconsistency between the main SEM and moderation test,

� measurement error not accounted for in interaction effects, and

� a risk of biased moderation estimates.

No test of measurement invariance or robustness.

Given the strong age skew and possible gender differences, the study does not test whether the measurement model is invariant across subgroups (e.g., male vs female, younger vs older). For a Q1 outlet, multi-group analysis or at least a discussion of this omission would be expected.

4. Data analysis and interpretation

Inconsistent description of hypothesis support.

The text states: “Paths 2, 3, 4 and 5 are significant, whereas paths 1 and 6 are not. Consequently, the model supports H2, H3, H4, H5 and H6.”

This is logically inconsistent: H6 explicitly hypothesises a non-significant effect, but the “support” is derived from a non-significant coefficient without proper equivalence testing. At minimum, the wording should distinguish “confirmed no significant effect” from “supported effect.”

Moderation result is statistically fragile.

The interaction term has β = –0.115 with a standard error of 0.129 according to Table 6, yet it is claimed as significant at 1%. A β smaller in magnitude than its standard error would normally not yield high significance; this suggests a possible typo or reporting error (in β, SE, or p‑value). A Q1 reviewer would ask for recalculation, reporting of t‑value, and perhaps the full regression output.

Effect sizes and practical significance are not reported.

R² values are given, but there is no discussion of effect sizes (e.g., standardized indirect effects, variance in loyalty explained uniquely by social value vs crowding) or whether differences are substantively meaningful.

No consideration of alternative models.

The model assumes a specific ordering: social value and crowding → experience quality → loyalty. However, plausible alternatives (e.g., experience quality influencing perceived social value; loyalty influencing intention to avoid hotspots) are not tested or discussed. A Q1 journal often expects at least a brief consideration of competing specifications.

5. Conceptual and empirical contradictions

Tension between high crowding perceptions and positive experience.

Descriptively, the item “The congestion in Tianshui has impacted my travel experience” has M = 3.37 (above midpoint) with high SD = 1.17, indicating many tourists felt impacted by congestion. Yet the discussion strongly emphasises that crowding “did not erode loyalty” and that congestion was “acceptable”. This interpretation is plausible but somewhat optimistic and not backed by segment-level analysis (e.g., high‑vs‑low crowding perception groups).

Claim that internet-famous status influences loyalty “only through indirect pathways” is overstated.

The study does not directly measure “internet-famous status” as a construct; rather, it uses perceived social value and perceived crowding as proxies, in a single destination already selected for being viral. To claim that “internet-famous destination status influences loyalty only through indirect pathways” extrapolates beyond what the data actually measure.

Quasi-experimental language without design.

The paper describes Tianshui as a “quasi-experimental” setting and references “viral cycles,” but there is no temporal or comparative design to justify this term. This can be perceived as overselling the methodological strength.

Other comments:

Figures not fully described: The text refers to Fig 1 (hypotheses model) and Fig 2 (estimated results) and lists them again as supporting information S1 and S2, but in the manuscript format shown, the figures are not visible and their content is only briefly mentioned. For review, paths, coefficients, and error terms in the figures should be clearly and consistently reported.

Ethical procedures described only briefly and without institutional approval information.

The paper notes verbal consent, anonymity, and parental consent for minors, but there is no mention of ethics committee approval or waiver, which many journals now require explicitly

Reviewer #3: Dear editor and authors:

I am very honoured for reviewing this paper, it has correct methods and good result. However, there has some questions you need to revise

1.Title: you'd have to add method used into your title, like""

2.Abstract: I suggest that all the highlights should be bolded and with the first letter capitalized. This will make it easier for readers to understand at a glance.

3.Keywords should be added into title page

4.The content of lines 178 to 194 is redundant. Delete this part or reduce it as much as possible to the description style of a scientific article. Now it seems more like a piece of news.

5.The entire "results" section should be a coherent whole, or it can be categorized using sub-sections or levels 2 and 3 headings. Descriptive statistics should include Table 1.

6.The first step in conducting a structural equation model should be the examination of common method variance to ensure the validity of the questionnaire. Please include this.

7.The "discussion" section should thoroughly analyze all the results. The length needs to be significantly increased. Currently, the entire article appears more like a draft rather than a complete paper.Maybe this paper will help you (10.1038/s41598-025-15612-6)

Reviewer #4: The manuscript titled *“Can Becoming an Internet-Famous Destination Influence Tourist Loyalty? An Investigation of Culinary Tourists in Tianshui, China”* addresses a timely and relevant topic by examining how perceived value shapes tourist loyalty in the context of social-media-driven destinations using structural equation modeling with 375 responses. The study demonstrates methodological rigor and offers useful insights; however, several issues must be addressed before it is suitable for publication. First, the theoretical contribution appears incremental, as relationships such as experience quality influencing loyalty are already well established; therefore, the authors should clearly articulate what is conceptually new—particularly whether the novelty lies in the “internet-famous destination” construct, the moderating role of comment interaction, or the interpretation of crowding effects. Relatedly, the term “internet-famous destination” requires a stronger academic definition with measurable attributes and clearer differentiation from popular or overtourism destinations. A major methodological concern is the use of a single-item measure for perceived comment interaction (“I think the comment section of the Tianshui Malatang short video is very interesting”), which raises questions about construct validity and should either be justified with literature or acknowledged as a limitation. The sample is heavily skewed toward young respondents aged 18–25 with relatively low income, potentially limiting generalizability; the authors should discuss generational bias and consider positioning the findings within Gen Z travel behavior. Additionally, because data were collected within a short holiday window using on-site questionnaires, the study captures loyalty intention rather than actual loyalty, and this distinction should be explicitly acknowledged along with the constraints of a cross-sectional design. The deletion of item X9 due to weak loading (0.445) is statistically acceptable but warrants further explanation regarding scale stability. The finding that crowding does not reduce loyalty is particularly interesting but under-theorized; expanding the discussion through concepts such as social proof, bandwagon effects, or FOMO could strengthen the contribution. From a presentation perspective, the manuscript would benefit from language polishing, reduced repetition (especially of “internet-famous destination”), stronger synthesis in the literature review rather than descriptive listing, and greater methodological transparency regarding random street intercept procedures and enumerator training. Practical implications should be expanded to provide more actionable recommendations for destination marketers, and the limitations section should be broadened to include the single-destination context, single-item moderator, cross-sectional data, and age-skewed sample. Overall, the study has clear publication potential due to its relevant topic, solid sample size, and robust statistical analysis, but substantial revision is necessary to strengthen theoretical positioning, measurement validity, and interpretation of findings.

Reviewer #5: Thank you for the opportunity to review this interesting manuscript related to loyalty in tourism, a very interesting theme. In my assessment, the paper is well structured and logically organised, which facilitates a clear understanding of the arguments presented. The theoretical framework is robust and grounded in relevant and up-to-date resources (academic literature). The purpose of the study is clearly presented in the abstract, and the selected keywords are appropriate and accurately reflect the theme of the research.

However, in my opinion, there are several issues that must be taken into account for the paper to be accepted for publication:

- the scientific novelty of the work should be presented in the abstract;

- the discussion section is poorly written; it should present the validation/invalidation of the established hypotheses and link them to the conclusions of relevant references;

- authors should clearly present the contribution of their research to the academic literature in the field and to the economic environment (stakeholders in the tourism sector).

Succes!

**********

what does this mean?). If published, this will include your full peer review and any attached files.

If you choose “no”, your identity will remain anonymous but your review may still be made public.

**Do you want your identity to be public for this peer review?**  For information about this choice, including consent withdrawal, please see our Privacy Policy

Reviewer #1: No

Reviewer #2: No

Reviewer #3: No

Reviewer #4: **Yes:** Dr.Shuvasree Banerjee

Reviewer #5: No

**********

[NOTE: If reviewer comments were submitted as an attachment file, they will be attached to this email and accessible via the submission site. Please log into your account, locate the manuscript record, and check for the action link "View Attachments". If this link does not appear, there are no attachment files.]

To ensure your figures meet our technical requirements, please review our figure guidelines: https://journals.plos.org/plosone/s/figures

You may also use PLOS’s free figure tool, NAAS, to help you prepare publication quality figures: https://journals.plos.org/plosone/s/figures#loc-tools-for-figure-preparation.

NAAS will assess whether your figures meet our technical requirements by comparing each figure against our figure specifications.

Attachment Submitted filename: Review of Manuscript BMC.pdf

# Author comment: Author response to Decision Letter 1

**DOI:** 10.1371/journal.pone.0357597.r002

14 Jun 2026

Response to Reviewers

1. Reviewer #1

Reviewers' comments:

Abstract

• Aim of the paper as captured in the abstract is completely different from the main titled

• The abstract did not contain any well-defined method

• The results presented are measuring associations without any proved of a known model fitted with the data captured in the abstract

• The conclusion or recommendations appeared as implications of a study which typically deviate from the traditional concept of abstract

Response

The title has been revised to "A Study of Tourist Loyalty at 'Internet-Famous' Destinations Based on Structural Equation Modeling: The Roles of Perceived Service Quality, Perceived Social Value, and Crowding Effects." This updated title more accurately reflects the research focus and the methodological framework.

Abstract Restructuring: The abstract has been rewritten to emphasize the application of Structural Equation Modeling (SEM). Furthermore, research inferences have been integrated with empirical measurement results to provide a more cohesive and comprehensive summary of the findings.

Correction of Causal Terminology: In alignment with scientific rigor for cross-sectional studies, all causal expressions (e.g., "influences," "leads to") have been replaced with relational or associational terminology (e.g., "is associated with," "is correlated with," or "predicts") to accurately reflect the links identified in the model.

Refinement of Expression: The overall phrasing and logical flow have been updated to ensure that the theoretical deductions are strictly supported by the measurement outcomes, thereby enhancing the clarity and consistency of the manuscript.

Reviewers' comments:

Introduction

• The introduction is generally okay

• But from 68-70, the main topic is different from the sentence such as “This paper seeks to investigate how varying levels of social media interaction contribute to differences in tourist loyalty, aiming to provide additional insights for social media marketing strategies in tourism destinations”

Response

Optimization of the Introduction: The introduction has been thoroughly restructured and optimized to establish a stronger logical connection with the revised title. Consequently, the previously mentioned text has been removed to ensure a more coherent and focused opening that better sets the stage for the research objectives.

Reviewers' comments:

Literature Review and Research Hypotheses

• The two concepts cannot be discussed together as captured

Research context

• I find it difficult to link why this section is placed where it is

Methodology

• The section is poorly written

• There are no sub headings explaining each concept and if possible justification for each

Response

Section Decoupling: The original section titled "Literature Review and Research Hypotheses" has been decoupled into two independent sections: "1. Research Framework" and "2. Research Hypotheses." This restructuring aims to provide a clearer distinction between the overarching theoretical logic and the specific path assumptions, thereby improving the manuscript's navigational clarity.

Removal of Research Background: The "Research Background" section has been removed to maintain a more concise and focused introduction.

Expansion of "Materials and Methods": The original "Materials and Methods" section has been expanded and restructured into three distinct subsections: "Study Area," "Data Collection," and "Variable Measurement." This restructuring provides a more granular and systematic description of the research methodology.

Optimization of Phrasing: The manuscript's overall phrasing has been refined for improved clarity and academic precision.

Expansion of "Variable Measurement": A dedicated "Variable Measurement" section has been added. In this section, we provide detailed explications of the conceptual connotations and the theoretical rationale for all variables used, ensuring that each construct is both well-defined and robustly justified within the research context.

Reviewers' comments:

Results

• Table 1, the use of “gender” is not appropriate as it explains the binary classification of the biological function of the respondents. Sex will be appropriate

• In Table 1, the classification of 51-60 years and 60 years and over should be checked

• The use of descriptive analysis in 222 as a sub heading is not appropriate since Table 1 is also descriptive in nature

• Table 2 position with the narrations should be checked.

• In Table 2 Measurement Reliability and Convergent Validity of the Scale, the emphasis on the Cronbach's α is not results but validation of the constructs to assess the internal consistency. Such is often placed in method section

• Chech where Table 3 is placed and the narration

Response

Refinement of Demographic Categories: In Table 1, the variable "Gender" has been replaced with "Sex" for biological accuracy. Additionally, the age category "60 and above" has been updated to "61 and above" to ensure precise, non-overlapping classification.

Subsection Reordering: The subheading "Descriptive Analysis" has been relocated to precede Table 1. The overall content of this section has been restructured to enhance the logical progression of the results.

Sequential Optimization of Tables: * Table 2 (Measurement Model results) has been repositioned immediately following the full-sample Confirmatory Factor Analysis (CFA) section.Table 3 has been placed following Table 2 to maintain a systematic presentation of the empirical evidence.

Methodological Enhancement: In the "Variable Measurement" subsection of the Methods, the results regarding Cronbach’s alpha coefficients have been integrated to provide early verification of the scales' reliability.

Reviewers' comments:

Hypothesis testing

• The nature of reporting this section is not clear

Discussion

• The entire section is done without a single reference to support the findings or disagree with the findings

• Candidate should revise to include references from the literature supporting the work or disagreeing with the study findings

Conclusion and Future Research

• The conclusion should be relooked at

• Including limitations in conclusion is not appropriate

Response

Restructuring of the Results Section: The Results section has been expanded into four distinct subsections to provide a more systematic presentation of the empirical findings: Path Analysis, Mediation Effect Testing, Moderation Effect Testing, and Supplementary Analysis.

Comprehensive Rewrite of the Discussion: The Discussion section has been entirely rewritten. We have incorporated extensive supporting evidence from the existing literature to contextualize our findings and provided more in-depth theoretical explanations for the observed relationships.

Update of Concluding Remarks: The Conclusions, Limitations, and Future Research sections have been rewritten to reflect the refined analytical results and to provide more nuanced insights for both theory and practice.

Reviewers' comments:

References

• The references should be formatted well

General comments

• Full stops on sub headings should be removed. Check 92, 110, 126 etc

• More work needs to be done on the manuscript

Response

Standardization of References: All references have been formatted strictly according to the journal's specific requirements.

Correction of Heading Formatting: The periods (full stops) following all subheadings have been removed .

Comprehensive Manuscript Review: A thorough, top-to-bottom revision of the entire manuscript has been conducted to ensure linguistic consistency, logical coherence, and formatting precision.

Reviewer #2:

Reviewers' comments:

1. Theoretical framing and hypotheses

Conceptualisation of “perceived social value” is narrow and overlaps with other constructs.

The scale items capture social attention, popularity, making friends, and self-confidence (X1–X4), which mix social status, social relationship gains, and self-related benefits under one factor, yet the theory section does not justify why these heterogeneous aspects belong to a single latent construct or how it differs from social image/value, self-enhancement, or hedonic value.

Operationalisation does not fully reflect SOR and UGT arguments.

Response

We reconstructed the "Perceived Social Value" construct based on the social value dimension of the PERVAL scale developed by Sweeney and Soutar (2001). Three indicators were retained: "This trip increases my social media followers," "This trip makes me more popular among friends," and "This trip helps me make more friends." The indicator "This trip makes me more independent and confident" was removed due to its conceptual ambiguity with social value. All indicators were renumbered accordingly, and the revised construct successfully passed the model validation.

Sweeney J C, Soutar G N. Consumer perceived value: The development of a multiple item scale. Journal of retailing, 2001, 77(2): 203-220.

Reviewers' comments:

Operationalisation does not fully reflect SOR and UGT arguments.

The SOR framing suggests multiple types of stimuli (UGC, authenticity of viral atmosphere, congruence between online and offline experience), but the model only includes perceived crowding and perceived social value as “stimuli”, with experience quality as “organism” and loyalty as “response”; key digital elements (e.g., online authenticity, congruence, expectations) are discussed conceptually but not measured.

Response

We have re-evaluated the theoretical framework of this study. The content related to Stimulus-Organism-Response (SOR) and Uses and Gratifications Theory (UGT) has been removed. Instead, we have restructured the research framework to focus more precisely on the interplay between perceived service quality and perceived social value, ensuring a more cohesive and streamlined logical flow.

Reviewers' comments:

H1 is not logically integrated with later mediation.

The hypothesis states that perceived social value directly increases loyalty (H1), then the mediation section argues that experience quality fully mediates this relationship and that social value has no direct effect on loyalty. This is not just a statistical outcome; it contradicts the initial claim that social value is a direct antecedent of loyalty, and there is no ex-ante rationale for why the effect should become fully indirect.

Response

Based on our literature review, existing research indicates that perceived social value is a precursor to loyalty, primarily exerting an indirect influence (Preko A., et al., 2020; Kim M., et al., 2018). Accordingly, the revised "Research Hypotheses" section clarifies this relationship and adopts a testing procedure that examines direct effects before proceeding to mediation analysis.

Preko A, Mohammed I, Ameyibor L E K. Muslim tourist religiosity, perceived values, satisfaction, and loyalty. Tourism Review International, 2020, 24(2-3): 109-125.

Kim M, Thapa B. Perceived value and flow experience: Application in a nature-based tourism context. Journal of destination marketing & management, 2018, 8: 373-384.

Reviewers' comments:

Contradiction in the treatment of H6.

H6 hypothesises “no significant influence” of perceived crowding on loyalty. The structural model finds a non-significant path (β = 0.044, p > 0.05) and labels H6 as “Accept.”

Accepting a null hypothesis is statistically contentious; usually one “fails to reject” it and would need power analysis or equivalence testing to support a claim of “no effect”. This should be reformulated or justified more rigorously.

Response

Refined Analytical Approach and Theoretical Integration: Following your constructive feedback, we have revised our testing methodology. Drawing on Stress Overload Theory, we initially hypothesized a negative impact of perceived crowding on tourist loyalty. Upon finding the linear hypothesis unsupported, we conducted a supplementary analysis to explore their underlying relationship. A quadratic regression model revealed a U-shaped non-linear relationship. We have interpreted this phenomenon through the lenses of Psychological Carrying Capacity, the Bandwagon Effect, and Fear of Missing Out (FOMO). These updates have been integrated into the "Research Hypotheses," "Hypothesis Testing," and "Discussion of Findings" sections.

Reviewers' comments:

Moderation by “perceived comment interaction” is under-theorised and minimally measured.

The moderation hypothesis (H7) is based on Uses-and-Gratifications Theory, but the moderator is measured by a single item (“I think the comment section … is very interesting”), which mainly reflects entertainment rather than interaction or participation (no frequency, depth, or type of engagement).

This weak alignment between theory (active co creation, gratifications) and measurement (interest in comments) raises construct validity concerns.

Response

We have repurposed the item "I find the comment section very interesting" to measure Perceived Playfulness. As hubs for secondary creation and Internet Memes, comment sections offer humor and social resonance that drive sensory satisfaction.

Furthermore, we have adopted Hedonic Declension Theory to explain the negative moderating effect of perceived playfulness on the relationship between service quality and loyalty. According to Wathieu (2004), consumers internalize past experiences as "reference points," requiring higher intensity or novelty to maintain satisfaction. The psychological response to repeated stimuli evolves through sensitization, habituation, and satiation. Galak (2018) and Chen (2019) suggest that repeated exposure generates negative cumulative effects; even satisfied customers may develop negative attitudes toward future consumption if they anticipate declining marginal utility.

In this study, we argue that while geographical distance once limited repetitive stimuli, short-video algorithms now enable high-frequency exposure to destination content. This compresses the cycle from sensitization to satiation. Pre-experiencing "viral" playful content leads to an early onset of hedonic declension, thereby weakening the formation of long-term loyalty and manifesting as a negative moderating effect.

Wathieu, L. Consumer habituation. Management Science, 2004,50(5), 587-596.

Becerril-Castrillejo, I., & Munoz-Gallego, P. A. Influence of habitual level of consumption on willingness to pay: A satiation, sensitization, and habituation approach. International Journal of Hospitality Management,2022, 103, 103210.

Galak, J., & Redden, J. P. The properties and antecedents of hedonic decline. Annual review of psychology, 2018, 69, 1-25.

Chen, C. C., & Liao, Y. S. From satisfaction to satiation: The gradation model of satiation in casual leisure. Annals of Leisure Research, 2019, 22(2), 233-246.

Reviewers' comments:

2. Research design and external validity

Single destination and very narrow time window.

Data are collected only in Tianshui during one holiday (Qing-Ming, 4–6 April 2024), within a specific boom period. This severely limits generalisability to other “internet-famous” destinations, off peak periods, or other types of viral events. The paper calls the setting a “quasi-experimental window” but does not employ any quasi-experimental design (no pre post, control group, or natural experiment structure).

Response

We fully acknowledge that data collected during a single holiday primarily reflects tourist experiences under peak conditions, which may limit the generalizability of the findings to off-peak periods. This limitation is now explicitly discussed in the "Research Limitations" section.

However, we contend that data captured during peak periods is uniquely suited to reflect tourist perceptions during viral tourism events. Since 2023, China has witnessed a surge in tourism phenomena characterized by short-video-driven, large-scale influxes of tourists in extremely short periods. This "online traffic-driven + offline surge" development model has emerged as a significant new pattern in Chinese tourism (Ye et al., 2026), warranting focused academic inquiry.

The data collection during the Qingming Festival (April 4–6) occurred at the peak of the destination’s popularity. Conclusions derived from this relatively

Attachment Submitted filename: Response to Reviewers.docx

# Peer review: Decision Letter 1

**DOI:** 10.1371/journal.pone.0357597.r003

**Authors:** Bo Pu

19 Jul 2026

Dear Dr. Huang,

Thank you for submitting your manuscript to PLOS ONE. After careful consideration, we feel that it has merit but does not fully meet PLOS ONE’s publication criteria as it currently stands. Therefore, we invite you to submit a revised version of the manuscript that addresses the points raised during the review process.

Please submit your revised manuscript by Sep 02 2026 11:59PM. If you will need more time than this to complete your revisions, please reply to this message or contact the journal office at plosone@plos.org. When you're ready to submit your revision, log on to https://www.editorialmanager.com/pone/ and select the 'Submissions Needing Revision' folder to locate your manuscript file.

- A letter that responds to each point raised by the academic editor and reviewer(s). You should upload this letter as a separate file labeled 'Response to Reviewers'.
- A marked-up copy of your manuscript that highlights changes made to the original version. You should upload this as a separate file labeled 'Revised Manuscript with Track Changes'.
- An unmarked version of your revised paper without tracked changes. You should upload this as a separate file labeled 'Manuscript'.

If you would like to make changes to your financial disclosure, please include your updated statement in your cover letter. Guidelines for resubmitting your figure files are available below the reviewer comments at the end of this letter.

If applicable, we recommend that you deposit your laboratory protocols in protocols.io to enhance the reproducibility of your results. Protocols.io assigns your protocol its own identifier (DOI) so that it can be cited independently in the future. For instructions see: https://journals.plos.org/plosone/s/submission-guidelines#loc-laboratory-protocols. Additionally, PLOS ONE offers an option for publishing peer-reviewed Lab Protocol articles, which describe protocols hosted on protocols.io. Read more information on sharing protocols at https://plos.org/protocols?utm_medium=editorial-email&utm_source=authorletters&utm_campaign=protocols.

As the corresponding author, your ORCID iD is verified in the submission system and will appear in the published article. PLOS supports the use of ORCID, and we encourage all coauthors to register for an ORCID iD and use it as well. Please encourage your coauthors to verify their ORCID iD within the submission system before final acceptance, as unverified ORCID iDs will not appear in the published article. *Only*  the individual author can complete the verification step; PLOS staff *cannot*  verify ORCID iDs on behalf of authors.

We look forward to receiving your revised manuscript.

Kind regards,

Bo Pu, Ph.D.

Academic Editor

PLOS One

Journal Requirements:

If the reviewer comments include a recommendation to cite specific previously published works, please review and evaluate these publications to determine whether they are relevant and should be cited. There is no requirement to cite these works unless the editor has indicated otherwise.

Please review your reference list to ensure that it is complete and correct. If you have cited papers that have been retracted, please include the rationale for doing so in the manuscript text, or remove these references and replace them with relevant current references. Any changes to the reference list should be mentioned in the rebuttal letter that accompanies your revised manuscript. If you need to cite a retracted article, indicate the article’s retracted status in the References list and also include a citation and full reference for the retraction notice.

Additional Editor Comments:

please revise this manuscript carefully according to the comments.

[Note: HTML markup is below. Please do not edit.]

Reviewers' comments:

Reviewer's Responses to Questions

**Comments to the Author**

Reviewer #1: All comments have been addressed

Reviewer #4: All comments have been addressed

Reviewer #5: All comments have been addressed

**********

2. Is the manuscript technically sound, and do the data support the conclusions?

Reviewer #1: Yes

Reviewer #4: Yes

Reviewer #5: Yes

**********

3. Has the statistical analysis been performed appropriately and rigorously?

Reviewer #1: Yes

Reviewer #4: Yes

Reviewer #5: Yes

**********

4. Have the authors made all data underlying the findings in their manuscript fully available?

The PLOS Data policy

Reviewer #1: Yes

Reviewer #4: Yes

Reviewer #5: Yes

**********

5. Is the manuscript presented in an intelligible fashion and written in standard English?

Reviewer #1: Yes

Reviewer #4: Yes

Reviewer #5: Yes

**********

Reviewer #1: REVIEW MANUSCRIPT DRAFT

Study on Tourist Loyalty at Internet-Famous Destinations Based on Structural Equation Modeling: Perceived Service Quality, Perceived Social Value, and Crowding Effects

Suggested title: Structural equation modeling of tourist loyalty in Tianshui, China: Perceived service quality, perceived social value, and crowding effects

ABSTRACT

• In line 24, delete (SEM)

• Use of “we” in line 24 should be delete

• There is no well defined method explained

• There is lack of stated findings

• This is not a discussion session to be drawing inferences

Introduction

• Provide reference to support the statement from line 39 to line 42

• From line 100 to line 106, no reference

Research Hypotheses

• I sincerely do not see the need to have a preamble which includes line 162 to line 174

• Again, from line 1774 to line 184

• Also, from line 187 to line 193

• From line 196 to line 209 is redundant

• From line 214 to line 221 is also not necessary

• From line 224 to 244 delete

• Having up to seven (7) hypothesis in one study is not an ideal thing to do. Kindly reduce the hypothesis to just two or at most three

• Again, the stated hypothesis, there are null or alternatives

Materials and methods

Data collection

• In line 274, the use of interviews is not appropriate with the study

• In line 279, the use of 375 valid questionnaires were recovered meant what?

• Provide a reference to support the range of value for the use of the Cronbach’s alpha coefficients. You may refer to Taber’s interpretations

Results

• In line 360, the use of Descriptive statistical analysis should be deleted

• In line 441, Table 2 should be moved to materials and methods if indeed it should be shown

• In Table 3, provide a legend for the acronyms used

Discussion

• In line 551, the discussion of findings should be deleted

Conclusion and Future Research

• In line 666, the heading should be deleted

General Comments

• There is significant improvement in the manuscript

• Authors may have a look at my humble suggestions to improve the content of the manuscript

Reviewer #4: The manuscript has undergone significant revision and demonstrates noticeable improvement. The responses to reviewers are comprehensive, and most of the previous concerns have been adequately addressed. The revised paper presents a clearer research framework, improved methodology, and stronger discussion. Nevertheless, several issues remain that should be addressed to further strengthen the manuscript.

Reviewer #5: The authors have made substantial improvements to the manuscript, and I consider it suitable for publication now. Good luck!

**********

what does this mean?). If published, this will include your full peer review and any attached files.

If you choose “no”, your identity will remain anonymous but your review may still be made public.

**Do you want your identity to be public for this peer review?**  For information about this choice, including consent withdrawal, please see our Privacy Policy

Reviewer #1: No

Reviewer #4: **Yes:** Dr.Shuvasree Banerjee

Reviewer #5: No

**********

[NOTE: If reviewer comments were submitted as an attachment file, they will be attached to this email and accessible via the submission site. Please log into your account, locate the manuscript record, and check for the action link "View Attachments". If this link does not appear, there are no attachment files.]

To ensure your figures meet our technical requirements, please review our figure guidelines: https://journals.plos.org/plosone/s/figures

You may also use PLOS’s free figure tool, NAAS, to help you prepare publication quality figures: https://journals.plos.org/plosone/s/figures#loc-tools-for-figure-preparation.

NAAS will assess whether your figures meet our technical requirements by comparing each figure against our figure specifications.

Attachment Submitted filename: REVIEW MANUSCRIPT DRAFT.pdf

# Author comment: Author response to Decision Letter 2

**DOI:** 10.1371/journal.pone.0357597.r004

18 Aug 2026

Response to Reviewers

1. Reviewer #1

Reviewers' comments:

Study on Tourist Loyalty at Internet-Famous Destinations Based on Structural Equation Modeling: Perceived Service Quality, Perceived Social Value, and Crowding Effects

Suggested title: Structural equation modeling of tourist loyalty in Tianshui, China: Perceived service quality, perceived social value, and crowding effects

Response：

The title of the manuscript has been revised to:Structural equation modeling of tourist loyalty in Tianshui, China: Perceived service quality, perceived social value, and crowding effects

Reviewers' comments:

ABSTRACT

• In line 24, delete (SEM)

• Use of “we” in line 24 should be delete

• There is no well defined method explained

• There is lack of stated findings

• This is not a discussion session to be drawing inferences

Response：

The abbreviation (SEM) has been removed, and the pronoun "we" has been eliminated throughout. We have comprehensively revised the abstract, provided clear descriptions of the research methodology, listed all empirical findings of this study, and removed analytical inferences within the abstract. Please refer to the revised manuscript for details.

Reviewers' comments:

Introduction

• Provide reference to support the statement from line 39 to line 42

• From line 100 to line 106, no reference

Response：

We have added the following references to support the arguments in Lines 39–42 of the manuscript：

Li K, Chen Z. From abroad to home: unveiling the surge in chinese domestic travel through the lens of affective economies. Journal of Tourism and Cultural Change, 2025, 23(4): 345-366.

In Lines 100–106 of the original manuscript, we introduce the Expectation-Confirmation Theory to reinterpret the effects of online information and offline experience on tourists’ perceptions, and two additional references are supplemented for theoretical support：

Oliver R L. A cognitive model of the antecedents and consequences of satisfaction decisions. Journal of marketing research, 1980, 17(4): 460-469.

Narangajavana Kaosiri Y, Callarisa Fiol L J, Moliner Tena M Á, et al. User-generated content sources in social media: A new approach to explore tourist satisfaction. Journal of Travel Research, 2019, 58(2): 253-265.

Reviewers' comments:

Research Hypotheses

• I sincerely do not see the need to have a preamble which includes line 162 to line 174

• Again, from line 1774 to line 184

• Also, from line 187 to line 193

• From line 196 to line 209 is redundant

• From line 214 to line 221 is also not necessary

• From line 224 to 244 delete

• Having up to seven (7) hypothesis in one study is not an ideal thing to do. Kindly reduce the hypothesis to just two or at most three

• Again, the stated hypothesis, there are null or alternatives

Response：

We sincerely appreciate the constructive comments from the reviewer. The reviewer suggested deleting all textual discussions in the "Research Hypotheses" section, retaining only the hypotheses, and consolidating the seven hypotheses into two to three. Following careful discussion and thorough deliberation regarding this suggestion, we respond as follows:

After thoroughly reviewing the narrative in the "Research Hypotheses" section, we found instances of textual redundancy and content overlap with the "Research Framework," which indeed required streamlining—likely the intended purpose of the reviewer's suggestion. However, the primary function of this section is to provide the theoretical basis for the causal paths between variables and to propose the research hypotheses for each path in the structural equation model. Deleting all introductory text would leave the hypothesized relationships lacking logical foundation, creating obstacles for readers to comprehend the paper. Therefore, we have substantially streamlined the narrative in the "Research Hypotheses" section by removing redundant wording and retaining only concise text to describe the theoretical derivation of the relationships, thereby ensuring structural integrity and readability.

The seven research hypotheses in this study correspond to seven distinct paths among variables in the structural equation model. Reducing the number of hypotheses to two or three would enhance conciseness and offer a quick overview of the study. However, doing so would cause each hypothesis to cover multiple paths belonging to different causal chains, potentially rendering the hypotheses ambiguous and hindering clear expression. To address the issue of having overly tedious hypotheses without losing clarity, we reorganized the original hypotheses into three logical categories and grouped them accordingly in the manuscript: (1) relationships among perceived service quality, perceived social value, and loyalty (H1–H3); (2) relationships between perceived crowding and the former three variables (H4–H6); and (3) the moderating effect of perceived playfulness (H7). This approach maintains the clarity of all seven paths while improving logical structure and reducing the reader's burden. Please refer to the revised manuscript for details.

Finally, all research hypotheses have been explicitly designated as alternative hypotheses.

Reviewers' comments:

Materials and methods

Data collection

• In line 274, the use of interviews is not appropriate with the study

• In line 279, the use of 375 valid questionnaires were recovered meant what?

Response：

The above comments were originally in the same paragraph; an overall adjustment has been made to this paragraph, correcting vague meanings and inappropriate phrasing. Please refer to the revised manuscript for details.

Reviewers' comments:

• Provide a reference to support the range of value for the use of the Cronbach’s alpha coefficients. You may refer to Taber’s interpretations

Response：

Added Taber's paper as supporting literature:

Taber K S. The use of Cronbach’s alpha when developing and reporting research instruments in science education. Research in science education, 2018, 48(6): 1273-1296.

Reviewers' comments:

Results

• In line 360, the use of Descriptive statistical analysis should be deleted

Response：

"Descriptive statistical analysis" has been removed.

Reviewers' comments:

• In line 441, Table 2 should be moved to materials and methods if indeed it should be shown

Response：

Regarding the section placement of Table 2, we have thoroughly reviewed and optimized it. Our response to this comment is as follows:

The metrics reported in the original Table 2 can be divided into three categories: descriptive statistics (the mean and SD for each item, representing the analysis of sample data); internal consistency test results (Cronbach’s α for each dimension, testing whether items under the same dimension are consistent in the basic data); and CFA results (standardized factor loadings for each item, as well as CR and AVE for each dimension, used to evaluate single-item convergence, overall dimensional consistency, and overall dimensional convergence). The original manuscript combined multiple types of metrics into a single table with the initial intention of saving space and avoiding duplicate tables, but this resulted in an overly cluttered table. However, moving Table 2 entirely to the "Materials and Methods" section would introduce a substantial amount of data analysis content into that section, blurring the distinct functions of each section, while also causing the CFA results to appear prematurely, thereby diminishing reading coherence.

Considering the above factors, we retained the location of Table 2 while streamlining its content by removing the Mean and SD columns, presenting text descriptions of the mean and SD for key metrics during the descriptive statistical analysis instead. This addresses the issue of Table 2 being overly cluttered without affecting the reporting of core data, while maintaining the coherence and logical rigor of the empirical testing in the "Results" section.

Reviewers' comments:

In Table 3, provide a legend for the acronyms used

Response：

All abbreviations in Table 3 have been changed to their full terms.

Reviewers' comments:

Discussion

• In line 551, the discussion of findings should be deleted

Response：

Content related to the study results has been deleted from the "Discussion" section.

Reviewers' comments:

Conclusion and Future Research

• In line 666, the heading should be deleted

Response：

the heading is already been deleted.

Reviewers' comments:

General Comments

• There is significant improvement in the manuscript

• Authors may have a look at my humble suggestions to improve the content of the manuscript

Response：

Thank you very much for your thorough and helpful comments. We have carefully considered all of your suggestions and revised the manuscript accordingly.

2. Reviewer #4

Reviewers' comments:

The manuscript has undergone significant revision and demonstrates noticeable improvement. The responses to reviewers are comprehensive, and most of the previous concerns have been adequately addressed. The revised paper presents a clearer research framework, improved methodology, and stronger discussion. Nevertheless, several issues remain that should be addressed to further strengthen the manuscript.

Response：

We greatly appreciate your positive evaluation of the revised manuscript. We have conducted a comprehensive self-examination and further polished the manuscript throughout the text to improve its quality.

As you mentioned that several issues remain to be addressed, we would highly appreciate it if you could provide specific comments on these remaining concerns. We are ready to make further targeted revisions strictly following your suggestions.

3. Reviewer #5

The authors have made substantial improvements to the manuscript, and I consider it suitable for publication now. Good luck!

Response：

Thank you very much for your positive assessment and recognition of our revisions. We highly value your time and efforts spent on this manuscript.

Attachment Submitted filename: Response_to_Reviewers_auresp_2.docx

# Editor report: Decision Letter 2

**DOI:** 10.1371/journal.pone.0357597.r005

**Authors:** Bo Pu

19 Aug 2026

Structural Equation Modeling of Tourist Loyalty in Tianshui, China: Perceived Service Quality, Perceived Social Value, and Crowding Effects

PONE-D-26-02054R2

Dear Dr. Huang,

We’re pleased to inform you that your manuscript has been judged scientifically suitable for publication and will be formally accepted for publication once it meets all outstanding technical requirements.

Within one week, you’ll receive an e-mail detailing the required amendments. When these have been addressed, you’ll receive a formal acceptance letter and your manuscript will be scheduled for publication.

An invoice will be generated when your article is formally accepted. Please note, if your institution has a publishing partnership with PLOS and your article meets the relevant criteria, all or part of your publication costs will be covered. Please make sure your user information is up-to-date by logging into Editorial Manager at Editorial Manager® and clicking the ‘Update My Information' link at the top of the page. For questions related to billing, please contact billing support.

If your institution or institutions have a press office, please notify them about your upcoming paper to help maximize its impact. If they’ll be preparing press materials, please inform our press team as soon as possible -- no later than 48 hours after receiving the formal acceptance. Your manuscript will remain under strict press embargo until 2 pm Eastern Time on the date of publication. For more information, please contact onepress@plos.org.

Kind regards,

Bo Pu, Ph.D.

Academic Editor

PLOS One

Additional Editor Comments (optional):

thanks for your hard work.

Reviewers' comments:

# Editor report: Acceptance letter

**DOI:** 10.1371/journal.pone.0357597.r006

**Authors:** Bo Pu

PONE-D-26-02054R2

PLOS One

Dear Dr. Huang,

I'm pleased to inform you that your manuscript has been deemed suitable for publication in PLOS One. Congratulations! Your manuscript is now being handed over to our production team.

At this stage, our production department will prepare your paper for publication. This includes ensuring the following:

* All references, tables, and figures are properly cited

* All relevant supporting information is included in the manuscript submission,

* There are no issues that prevent the paper from being properly typeset

You will receive further instructions from the production team, including instructions on how to review your proof when it is ready. Please keep in mind that we are working through a large volume of accepted articles, so please give us a few days to review your paper and let you know the next and final steps.

Lastly, if your institution or institutions have a press office, please let them know about your upcoming paper now to help maximize its impact. If they'll be preparing press materials, please inform our press team within the next 48 hours. Your manuscript will remain under strict press embargo until 2 pm Eastern Time on the date of publication. For more information, please contact onepress@plos.org.

You will receive an invoice from PLOS for your publication fee after your manuscript has reached the completed accept phase. If you receive an email requesting payment before acceptance or for any other service, this may be a phishing scheme. Learn how to identify phishing emails and protect your accounts at https://explore.plos.org/phishing.

If we can help with anything else, please email us at customercare@plos.org.

Thank you for submitting your work to PLOS One and supporting open access.

Kind regards,

PLOS One Editorial Office Staff

on behalf of

Dr. Bo Pu

Academic Editor

PLOS One
